# Selective and Controlled Grafting from PVDF-Based Materials by Oxygen-Tolerant Green-Light-Mediated ATRP

**DOI:** 10.1021/acsami.4c03369

**Published:** 2024-04-23

**Authors:** Piotr Mocny, Ting-Chih Lin, Rohan Parekh, Yuqi Zhao, Marek Czarnota, Mateusz Urbańczyk, Carmel Majidi, Krzysztof Matyjaszewski

**Affiliations:** † Department of Chemistry, Carnegie Mellon University, 4400 Fifth Ave., Pittsburgh, Pennsylvania 15213, United States; ‡ Faculty of Chemistry, University of Warsaw, Pasteura 1, 02-093 Warsaw, Poland; § Department of Materials Science & Engineering, Carnegie Mellon University, 5000 Forbes Ave., Pittsburgh, Pennsylvania 15213, United States; ∥ Department of Mechanical Engineering, Carnegie Mellon University, 5000 Forbes Ave., Pittsburgh, Pennsylvania 15213, United States; ⊥ Institute of Physical Chemistry, Polish Academy of Sciences, Kasprzaka 44/52, 01-224 Warsaw, Poland

**Keywords:** poly(vinylidene fluoride), fluoropolymers, ATRP, photopolymerization, grafting, DOSY NMR, stretchability, toughness

## Abstract

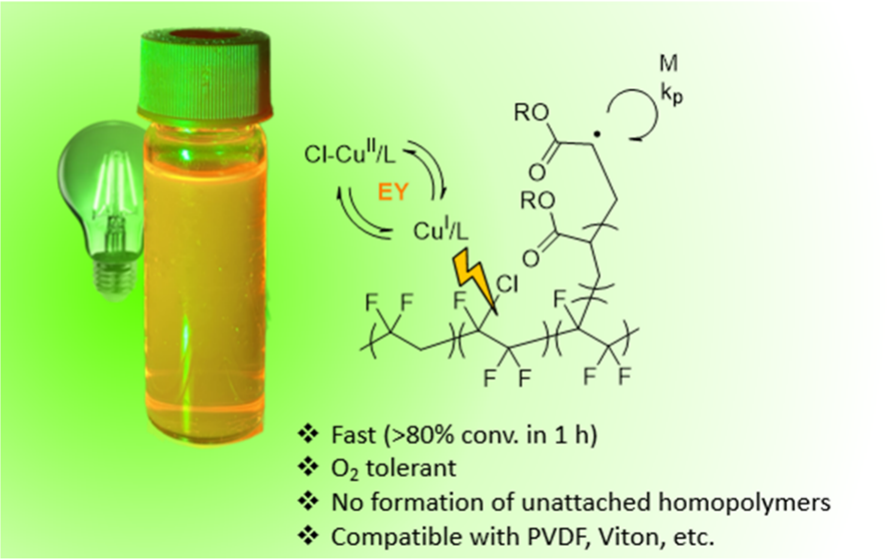

Poly(vinylidene fluoride) (PVDF) shows excellent chemical and thermal resistance and displays high dielectric strength and unique piezoelectricity, which are enabling for applications in membranes, electric insulators, sensors, or power generators. However, its low polarity and lack of functional groups limit wider applications. While inert, PVDF has been modified by grafting polymer chains by atom transfer radical polymerization (ATRP), albeit via an unclear mechanism, given the strong C–F bonds. Herein, we applied eosin Y and green-light-mediated ATRP to modify PVDF-based materials. The method gave nearly quantitative (meth)acrylate monomer conversions within 2 h without deoxygenation and without the formation of unattached homopolymers, as confirmed by control experiments and DOSY NMR measurements. The gamma distribution model that accounts for broadly dispersed polymers in DOSY experiments was essential and serves as a powerful tool for the analysis of PVDF. The NMR analysis of poly(methyl acrylate) graft chain-ends on PVDF-CTFE (statistical copolymer with chlorotrifluoroethylene) was carried out successfully for the first time and showed up to 23 grafts per PVDF-CTFE chain. The grafting density was tunable depending on the solvent composition and light intensity during the grafting. The initiation proceeded either from the C–Cl sites of PVDF-CTFE or via unsaturations in the PVDF backbones. The dehydrofluorinated PVDF was 20 times more active than saturated PVDF during the grafting. The method was successfully applied to modify PVDF, PVDF-HFP, and Viton A401C. The obtained PVDF-CTFE-*g*-PnBMA materials were investigated in more detail. They featured slightly lower crystallinity than PVDF-CTFE (12–18 vs 24.3%) and had greatly improved mechanical performance: Young’s moduli of up to 488 MPa, ductility of 316%, and toughness of 46 × 10^6^ J/m^3^.

## Introduction

Poly(vinylidene fluoride) (PVDF)-based materials are attractive due to their high chemical and thermal stability, as well as mechanical performance.^1^ They display unique dielectric, piezoelectric, and ferroelectric properties^2^ that make them well suited for applications in separation membranes,^3^ binders,^4,5^ separators,^6,7^ and electrolytes^8^ in batteries, as well as sensors,^9,10^ actuators,^9^ and energy generators.^11^ However, progress in the use of PVDF in emerging application domains is currently hindered by several bottlenecks. Unmodified PVDF membranes display limited wetting, which is very disadvantageous for mass transport.^3^ The chemical inertness of PVDF, while beneficial for its stability, poses limitations for effective binding in battery applications.^4^ Furthermore, the dielectric behavior of PVDF, which is essential for sensors and actuators, is highly dependent on the synthetic procedure. Polymerization of vinylidene fluoride (VDF) proceeds with the generation of head-to-head (HH) and tail-to-tail (TT) defects, which are unavoidable, but their number can vary and have a huge effect on chain conformation and polymorphic forms. For example, above 11 mol % of these defects, normal ferroelectric β-phase of PVDF dominates.^12^ The crystal structure may be further adjusted by the copolymerization of vinylidene fluoride (VDF) with other monomers, such as trifluoroethylene (TFE) or chlorotrifluoroethylene (CTFE).^13^ Nevertheless, these copolymerizations are not trivial to conduct, as they usually require high-pressure steel autoclaves to hold gaseous monomers (bp_VDF_ = −84 °C, bp_TFE_ = −51 °C, bp_CTFE_ = −28 °C) at elevated temperatures (100–250 °C).^14^

For these reasons, direct adjustment of the properties of PVDF by simple chemical modification is desired with two approaches typically used. The grafting-onto strategy utilizes the tendency of PVDF to dehydrofluorinate under basic conditions. The generated unsaturations are targeted by radical additions.^15−19^ PVDF may also be preactivated to introduce functional groups for subsequent grafting, e.g., ozone treatment to generate hydroxyl groups followed by Steglich esterification.^20^ The second approach, grafting-from PVDF, can proceed by abstracting hydrogen atoms from PVDF by radical initiators^21^ or by abstracting fluorine atoms in atom transfer radical polymerization (ATRP).^22−33^ ATRP is one of the controlled radical polymerization techniques that relies on a reversible deactivation process to synthesize polymer chains with predetermined molecular weight and low dispersity (*Đ*).^34−39^ A catalyst, typically a copper complex with a polydentate amine ligand (L) in its lower oxidation state (Cu(I)/L), abstracts a halogen atom from an alkyl halide initiator (R-X) or a dormant polymer chain-end (P*_n_*-X) to create a propagating radical. This radical is subsequently deactivated in a reverse reaction with a catalyst at a higher oxidation state (X-Cu(II)/L). This activation–deactivation equilibrium enables the concurrent growth of polymer chains as well as the preservation of the halide chain-end functionality. The exact mechanism of the Cu-mediated ATRP grafting-from PVDF is, however, debated, as homolytic cleavage of a regular C–F bond is unrealistic due to its high bond dissociation energy (BDE_C–F_ = 116 kcal/mol, compared with BDE_C–Cl_ = 78.5 kcal/mol and BDE_C–H_ = 98.8 kcal/mol).^40^ Hydrogen abstraction is, in principle, also possible. It seems that in certain configurations, e.g., at HH defects of PVDF, i.e., CH_2_**-CF**_**2**_**-CF**_**2**_**-**CH_2_, some carbon−fluorine bonds are easier to cleave.^41,42^ Some explanations involve the preceding formation of double bonds, which may lower the BDE of adjacent C–F bonds. Indeed, allyl halides are efficient ATRP initiators^43^ with activity similar to methacrylic halides (such as α-haloisobutyrates commonly used in ATRP, compare Δ*G*°_298,allyl-Cl_ = 55.8 kcal/mol, Δ*G*°_298,MMA-Cl_ = 57.4 kcal/mol, calculated by density functional theory (DFT)^44^). These double bonds may also participate in radical addition reactions and the modification may proceed via the grafting-through mechanism.^40^ This is often deliberately exploited to modify PVDF membranes, which are pretreated with a base and are conveniently performed in a solid state. The CH=CF units of dehydrofluorinated PVDF have nevertheless low reactivity toward radical addition due to the strong electron-withdrawing property of the F atom and improved grafting is obtained through CH=CH units in a specially synthesized PVDF variant.^45^ Alternatively, radiation-induced graft polymerization,^46−48^ polymerization through physisorbed radical initiators,^21^ polymerization from oxygen plasma-activated PVDF,^49^ or ozone-treated dissolved PVDF may be used.^50,51^

Because the mechanism of the direct grafting from PVDF by ATRP is unclear, copolymers of VDF with chlorotrifluoroethylene, CTFE, (P(VDF-*co*-CTFE)) bearing weaker C–Cl bonds (BDE_C–Cl_ = 78.5 kcal/mol), are used for a more defined modification.^30^ Most of the ATRP protocols, however, utilize high amounts of copper catalysts and ligands, which increases synthetic costs and introduces contamination and coloration of the product.^28,30,52−56^ As bases, these ligands may also cause dehydrofluorination.^40^ Recently, a UV-mediated ATRP from P(VDF-*co*-CTFE) was reported,^29^ using as low as 195 ppm of copper reaching 25% conversion in 6 h. The authors also observed the unwanted formation of unattached homopolymers. In another report, an expensive iridium-based photoredox complex, *fac*-[Ir(ppy)_3_], was used for visible-light-mediated ATRP.^23^ A more challenging P(VDF-*co*-TFE) polymer was used, and the grafting efficiencies were relatively low, i.e., up to 12.2%. Recent reports by Loos et al. explored metal-free photomediated ATRP to obtain PVDF-based block copolymers and raised importance of avoiding residual copper in these materials for biomaterials and microelectronics.^57,58^

**Scheme 1:**
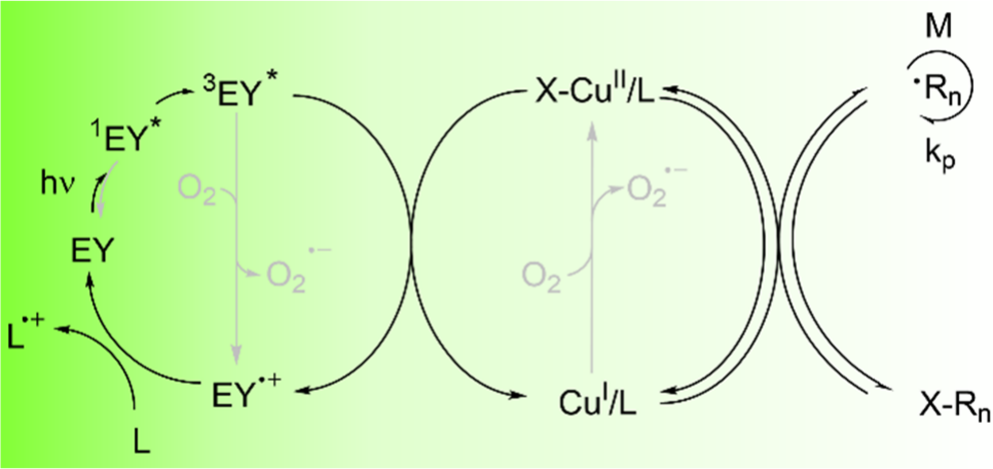
Mechanism of EY/Cu-Catalyzed ATRP^60−62^

Here, we utilize green-light-driven ATRP for grafting-from PVDF-CTFE using inexpensive eosin Y (EY) as a photocatalyst. We have recently applied it to modify NCM811 cathode binders, but without focus on synthetic conditions and its consequences.^59^ Eosin Y was selected over other photocatalysts, such as *fac*-[Ir(ppy)_3_] which operate under higher-energy light and are also more prone to side reactions. The method, as described in previous reports,^60−62^ represents a dual-catalytic approach whereupon photoexcitation, eosin Y reduces copper complex to its activator form, which activates/initiates polymerization (Scheme 1). This process is oxygen-tolerant and is particularly efficient in polar media, which enables high monomer conversions in a short time. Low-energy green light, ppm level of copper catalyst, and temporally controlled polymerization are key to selective and clean process, where the formation of unattached homopolymers, as well as unwanted ligand-mediated elimination (dehydrochlorination, dehydrofluorination) can be avoided.

## Experimental Section

### Materials

All chemicals were purchased from commercial sources and used as received, unless stated otherwise. Tris[2-(dimethylamino)ethyl]amine (Me_6_TREN, 99%)CuCl_2_ was purchased from Ambeed. Eosin Y disodium salt (Acid Red 87, >90%) was purchased from TCI. Copper(II) bromide (CuBr_2_, 99.99%), copper(II) chloride (CuCl_2_, 99.99%), poly(vinylidene fluoride) (PVDF, average *M*_w_ = 275,000, *M*_n_ = 107,000), and poly(vinylidene fluoride-*co*-hexafluoropropylene) (PVDF-HFP, average *M*_w_ = 400,000, *M*_n_ = 130,000) were purchased from Sigma-Aldrich. Poly(vinylidene fluoride-*co*-chlorotrifluoroethylene) (PVDF-CTFE, 90/10 wt %, *M*_w_ = 292,000, *M*_n_ = 108,000, gel permeation chromatography-multiangle light scattering (GPC-MALS) with DMF as an eluent) was purchased from PolyK. Dehydrofluorinated poly(vinylidene fluoride) (DHF-PVDF, 9 mol %) was kindly supplied by the group of Prof. Henry Sodano from University of Michigan. Viton A401C was kindly supplied by Woodward, Inc. (original supplier; Chemours). Methyl acrylate (MA, 99%), *tert*-butyl acrylate (tBA, >99%), *n*-butyl acrylate (nBA, >99%), *n*-butyl methacrylate (nBMA, 99%), poly(ethylene glycol) methyl ether methacrylate (PEGMEA, average *M*_n_ = 480 g/mol, >99%), and lauryl acrylate (LA, 90%) were purchased from Sigma-Aldrich. 2-Cyanoethyl acrylate (CEA, >95%) was purchased from TCI. The inhibitor from the monomers was removed by passing through a plug with activated basic alumina. Dimethyl sulfoxide (DMSO, >99.9%), methanol (99.8%), and acetone (99.5%) were purchased from Fisher Chemical. Dimethylformamide (DMF, >99.5%) was purchased from TCI. Anisole. Deionized water was obtained from Carnegie Mellon facilities. Methyl isobutyl ketone (MIBK, 99%) and methyl ethyl ketone (MEK, 99%) were purchased from Sigma-Aldrich.

## Procedures

### Polymerizations

Polymerizations of MA, nBA, and nBMA reported in the main text were carried out in the EvoluChem PhotoRedOx Box device purchased from HepatoChem equipped with green LEDs (λ = 525 nm, Kessil) (Figure S1). The stirring was fixed at 300 rpm throughout all experiments. The light intensity was tuned between 25 and 100% (25 mW/cm^2^ at 100%). The polymerizations were carried out in clear 1-dram vials (12/96 mm). No air cooling was provided; however, temperatures never exceeded 30 °C in this setup. The polymerizations of other monomers (tBA, PEGMEA, CEA, and LA) were carried out in homemade photoreactors using 20 mL clear glass vials. These photoreactors were prepared by wrapping green LED strips (aspectLED, 525 nm) around a crystallizing dish and shielding it with aluminum foil. The light exposure in this setup was 26 mW/cm^2^, and temperatures were reaching 60 °C. The light exposures were measured by an optical power meter (PM100D, ThorLabs) with a light sensor (S120VC, 200–1100 nm, 50 mW).

#### Synthesis of PVDF-CTFE-*g*-PMA

First, 0.466 g of PVDF-CTFE (90/10 wt %) was placed in a 1-dram vial with a stir bar. 2.936 mL of DMSO and 63.3 μL of DMF were added. The vial was closed, heated, and mixed until complete dissolution of the polymer. Subsequently, 0.72 mL of MA (0.689 g, 8 mmol), 43 μL of 5 mg/mL CuCl_2_ stock solution in DMF (0.22 mg, 1.6 μmol), 55.3 μL of 20 mg/mL Me_6_TREN stock solution in DMF (1.11 mg, 4.8 μmol), and 110.7 μL of 3 mg/mL eosin Y disodium salt stock solution in DMSO (0.33 mg, 0.48 μmol) were added. The resulting mixture was mixed thoroughly, placed in a PhotoRedOx Box, and irradiated under a given light intensity (25, 50, or 100%) for a desired time (typically 8 h for 25% and 1 h for 100% light intensity). Aliquots were taken and used to determine monomer conversion (NMR) and follow the molecular weight distribution (GPC). The temperature within the photoreactor chamber was measured every 15 min using an infrared thermometer to evaluate an average. The procedure represents an experiment with the following conditions: ratio of MA/C–Cl/CuCl_2_/Me_6_TREN/EY·Na_2_ = 20/1/0.004/0.012/0.0012, [CuCl_2_]/[MA] = 200 ppm, 3 equiv of Me_6_TREN, 0.3 equiv of EY·Na_2_, [MA] = 2 M, *V*_tot_ = 4 mL, MA/PVDF-CTFE = 1.48/1 w/w. Other experiments were scaled accordingly.

#### Synthesis of PVDF-CTFE-*g*-PnBA

Three ratios of nBA/C–Cl were used: 20/1, 50/1, and 200/1 (nBA/PVDF-CTFE = 2.2/1, 5.51/1, and 22.05 w/w). First, 1.864 g (nBA/C–Cl = 20/1), 0.745 g (nBA/C–Cl = 50/1), or 0.186 g (nBA/C–Cl = 200/1) of PVDF-CTFE (90/10 wt %) was placed into a 20 mL glass vial with a stir bar. Then, 2.14 mL of DMSO and 8.75 mL of DMF were added. The vial was closed with a rubber septum, heated, and mixed until complete dissolution of the polymer. Subsequently, 4.57 mL of nBA (32 mmol), 172 μL of 5 mg/mL CuCl_2_ stock solution in DMF (0.86 mg, 6.4 μmol), 221 μL of 20 mg/mL Me_6_TREN stock solution in DMF (4.42 mg, 19.2 μmol), and 148 μL of 3 mg/mL eosin Y disodium salt stock solution in DMSO (0.44 mg, 0.64 μmol) were added. The resulting mixture was mixed thoroughly, placed in a PhotoRedOx Box, and irradiated under 100% light intensity until desired conversion was reached. Aliquots were taken and used to determine monomer conversion (NMR) and follow molecular weight distribution (GPC). Ratios of nBA/C–Cl/CuCl_2_/Me_6_TREN/EY·Na_2_ = 20/1/0.004/0.012/0.0004, 50/1/0.01/0.03/0.001, [CuCl_2_]/[nBA] = 200 ppm, 3 equiv of Me_6_TREN, 0.1 equiv of EY·Na_2_, [nBA] = 2 M, *V*_tot_ = 16 mL, nBA/PVDF-CTFE = 2.2/1, 5.51/1, and 22.05 w/w.

#### Synthesis of PVDF-CTFE-*g*-PnBMA

Three ratios of nBMA/C–Cl were used: 20/1, 50/1, and 200/1 (nBMA/PVDF-CTFE = 2.44/1, 6.11/1, and 24.46 w/w). First, 1.864 g (nBMA/C–Cl = 20/1), 0.745 g (nBMA/C–Cl = 50/1), or 0.186 g (nBMA/C–Cl = 200/1) of PVDF-CTFE (90/10 wt %) was placed into a 20 mL glass vial with a stir bar. Then, 2.03 mL of DMSO and 8.33 mL of DMF were added. The vial was closed with a rubber septum, heated, and mixed until the polymer. Subsequently, 4.55 mL of nBA (32 mmol), 172 μL of 5 mg/mL CuCl_2_ stock solution in DMF (0.86 mg, 6.4 μmol), 221 μL of 20 mg/mL Me_6_TREN stock solution in DMF (4.42 mg, 19.2 μmol), and 148 μL of 3 mg/mL eosin Y disodium salt stock solution in DMSO (0.44 mg, 0.64 μmol) were added. The resulting mixture was mixed thoroughly, placed in a PhotoRedOx Box, and irradiated under 100% light intensity for 80 min until the desired conversion was reached. Aliquots were taken and used to determine monomer conversion (NMR) and follow molecular weight distribution (GPC). Ratios of nBMA/C–Cl/CuCl_2_/Me_6_TREN/EY·Na_2_ = 20/1/0.004/0.012/0.0004, 50/1/0.01/0.03/0.001, [CuCl_2_]/[nBA] = 200 ppm, 3 equiv of Me_6_TREN, 0.1 equiv of EY·Na_2_, [nBA] = 2 M, *V*_tot_ = 16 mL, nBMA/PVDF-CTFE = 2.44/1, 6.11/1, and 24.46 w/w.

#### Synthesis of PVDF-CTFE-*g*-PtBA (Table S1, Figures S22 and S23)

Three ratios of tBA/C–Cl were used: 20/1, 30/1, and 80/1 (tBA/PVDF-CTFE = 2.20/1, 4.40/1, and 8.80 w/w). First, 1.2 g (tBA/C–Cl = 20/1), 0.6 g (tBA/C–Cl = 40/1), or 0.3 g (tBA/C–Cl = 80/1) of PVDF-CTFE (90/10 wt %) was placed into a 20 mL glass vial with a stir bar. Then, 4.01 mL of anisole and 5.77 mL of DMF were added. The vial was closed with a rubber septum, heated, and mixed until complete dissolution of the polymer. Subsequently, 3.02 mL of tBA (20.6 mmol), 111 μL of 5 mg/mL CuCl_2_ stock solution in DMF (0.554 mg, 4.12 μmol), 142 μL of 20 mg/mL Me_6_TREN stock solution in DMF (2.85 mg, 12.4 μmol), and 95 μL of 3 mg/mL eosin Y disodium salt stock solution in DMSO (0.29 mg, 0.41 μmol) were added. The resulting mixture was mixed thoroughly, placed in a homemade photoreactor, and irradiated under green light intensity (aspectLED) for a given time until desired conversion was reached. Note that this setup resulted in heating to 60 °C, which facilitated the reaction in the anisole/DMF solvent. Aliquots were taken and used to determine monomer conversion (NMR) and follow molecular weight distribution (GPC). Ratios of tBA/C–Cl/CuCl_2_/Me_6_TREN/EY·Na_2_ = 20/1/0.004/0.012/0.0004, 40/1/0.008/0.024/0.0008, [CuCl_2_]/[tBA] = 200 ppm, 3 equiv of Me_6_TREN, 0.1 equiv of EY·Na_2_, [tBA] = 1.57 M, *V*_tot_ = 13.15 mL, 2.20/1, 4.40/1, and 8.80 w/w.

#### Synthesis of PVDF-CTFE-*g*-PEGMEA (Procedure a, Table S2)

PEGMEA/Cl = 40/1. In a 20 mL vial, 0.6 g (0.515 mmol) of PVDF-CTFE (C–Cl) was dissolved in 6 mL of DMF and 0.8 mL of DMSO. Then, 9.07 mL (9.888 g, 0.0206 mol) of PEGMEA, 110.8 μL (0.00412 mmol, 5 mg/mL solution in DMF) of CuCl_2_, 95 μL (0.412 μmol, 3 mg/mL solution in DMSO) of eosin Y disodium salt, and 142.4 μL (0.0124 mmol, 20 mg/mL solution in DMF) of Me_6_TREN were added to the vial. It was then covered with a rubber stopper, and the reaction mixture was degassed by nitrogen bubbling for 10 min. The vial was then placed under a green light aspectLED in a homemade photoreactor. Once the reaction was complete (conversion ∼50%), the mixture was dialyzed against methanol. Other polymerizations from Table S2, i.e., ratios of PEGMEA/Cl = 20/1, 5/1, and 2.5/1 were scaled accordingly.

#### Synthesis of PVDF-CTFE-*g*-PLA (LA, Table S2)

Stock solutions of 5 mg/mL CuCl_2_ and 20 mg/mL Me_6_TREN were prepared in DMF. A stock solution of 3 mg/mL eosin Y disodium salt was prepared in DMSO. Into a 10 mL Schlenk flask were added PVDF-CTFE (0.4 g, 0.34 mmol, 1 equiv), 5.3 mL of DMF, and 0.70 mL of DMSO. The solution was heated gently in a 50 °C oil bath to dissolve PVDF-CTFE completely. LA (3.3 g, 13.7 mmol, 40 equiv), CuCl_2_ (0.74 mg, 5.5 μmol, 0.016 equiv), Me_6_TREN (3.8 mg, 16.5 μmol, 0.048 equiv), and eosin Y (0.38 mg, 0.55 μmol, 0.0016 equiv) stock solutions were then added to the flask. The flask was sealed and purged with nitrogen for 20 min. The reaction was irradiated under green light (aspectLED, homemade photoreactor) to begin the reaction. After 150 min, the reaction was purged by opening the flask to the atmosphere.

#### Synthesis of PVDF-CTFE-*g*-P(PEGMEA-*co*-tBA) (Procedure 3a, Table S3)

PEGMEA/Cl = 20/1; tBA/Cl = 20/1. In a 20 mL vial, 0.6 g (0.515 mmol) of PVDF-CTFE was dissolved in 6 mL of DMF and 0.8 mL of DMSO. Then, 4.54 mL (4.944 g, 0.0103 mol) of PEGMEA 1.51 mL (1.32 g, 0.0103 mol) of tBA, 110.8 μL (0.00412 mmol, 5 mg/mL solution in DMF) of CuCl_2_, 95 μL (0.412 μmol, 3 mg/mL solution in DMSO) of eosin Y, and 142.4 μL (0.0124 mmol, 20 mg/mL solution in DMF) of Me_6_TREN were added to the vial. The vial was then covered with a rubber stopper, and the reaction mixture was degassed by nitrogen bubbling for 10 min. It was then placed under a green light lamp at room temperature. Once the reaction was complete (conversion ∼50%), the mixture was dialyzed in methanol. Other polymerizations from Table S3, i.e., ratios of PEGMEA/Cl = 2.5/1, tBA/Cl = 17.5/1, and PEGMEA/Cl = 5.9/1, tBA/Cl = 16.1 were scaled accordingly.

#### Synthesis of PVDF-CTFE-*g*-P(PEGMEA-*co*-CEA) (Procedure 2, Table S2)

PEGMEA/Cl = 9.8/1, and CEA/Cl = 15/1. In a 20 mL vial, 0.6 g (0.000515 mol) of PVDF-CTFE (90/10 wt %) was dissolved in 6 mL of DMF and 0.8 mL of DMSO. Then, 2.21 mL (2.41 g, 0.0050 mol) of PEGMEA, 0.688 mL (0.967 g, 0.0077 mol) of CEA, 68.6 μL (0.00255 mmol, 5 mg/mL solution in DMF) of CuCl_2_, 58.8 μL (0.255 μmol, 3 mg/mL solution in DMSO) of eosin Y and 88.1 μL (0.00765 mmol, 20 mg/mL solution in DMF) of Me_6_TREN were added to the vial. The vial was then covered with a rubber stopper and the reaction mixture was degassed by nitrogen bubbling for 10 min. It was then placed under a green light (aspectLED) in a homemade photoreactor. Once the reaction was complete (conversion ∼12.5%), the mixture was dialyzed in methanol. Procedure 3 from Table S2 was scaled accordingly.

#### Solvent casting

The polymers were dissolved in DMF (40 mg/mL) at 60 °C and were cast into 15 mm × 5 mm rectangular Teflon molds. After most of the solvent slowly evaporated over 24 h at room temperature, molds were transferred to a vacuum oven and continuously evaporated at 120 °C for 48 h. Free-standing films with thicknesses of 100–200 μm were obtained.

## Analytical Methods

### Nuclear Magnetic Resonance (NMR) Spectroscopy

^1^H NMR spectra were recorded on a Bruker Avance III 500 MHz equipped with a multinuclear BBFO plus smart room temperature probe (16 scan monomer conversion measurements) or NEO 500 MHz equipped with a multinuclear prodigy cryoprobe (256 scan chain-end analyses). DMSO-*d*_6_ or acetone-*d*_6_ was used as a solvent depending on the polarity of the grafts.

### Diffusion-Ordered NMR Spectroscopy (DOSY NMR)

For the PVDF-CTFE-*g*-PMA, the DOSY NMR experiments were carried out on a NEO 500 MHz machine. The standard Bruker pulse program, ledbpgp2s, employing longitudinal eddy current delay (LED) with bipolar gradient pulse pair, 2 pulse gradients, was used. Diffusion delay, Δ = 400 ms (d20 = 0.4 s), and diffusion gradient pulse length, δ = 4 ms (p30 = δ/2 = 2000 μs), were used unless stated otherwise.

For other samples, the DOSY experiments were carried out on Bruker Avance II equipped with a Diff30 probe. The measurements were performed by using a series of stebpgp1s1d sequences. To ensure good SNR during the whole acquisition, the number of scans was increased from 16 for low power gradient pulses to 256 for higher gradients. This method approach is an adaptation of matched accumulation^1^ for diffusion measurement. The diffusion delay, Δ = 100 ms (d20 = 0.1 s), and diffusion gradient pulse length, δ = 8 ms (p30 = δ/2 = 4000 μs), were used unless stated otherwise.

Both pulse sequences are suitable for conditions where convection currents are not expected, such as in DMSO-*d*_6_. Typically, 10 mg/mL samples in deuterated solvents were prepared.

All data was processed using Python 3 script using nmrglue, numpy, scipy, and matplotlib libraries. For the PVDF-CTFE-*g*-PMA sample, the whole processing was done using nmrglue to obtain spectra, and others were imported as spectra already processed in TOPSPIN 4.2.0. Then, regions for integration were chosen. For samples measured on a Bruker Avance II, each integral was divided by the corresponding number of scans. Then, integrals were normalized by dividing all by the value of the integral of the first spectrum. From normalized integrals, the mean diffusion coefficient and standard deviation parameters were obtained after fitting with the gamma distribution model.

### Gel Permeation Chromatography (GPC)

GPC measurements of polymers were performed using Agilent GPC equipped with RI detector and PSS columns (Styrogel 10^5^, 10^3^, 10^2^ Å) with DMF as an eluent at 50 °C and the flow rate of 1 mL/min. Linear poly(methyl methacrylate) standards were used for calibration. Alternatively, measurements were performed using Agilent GPC equipped with an RI detector and PSS columns (SDV 10^3^, 10^5^, and 10^6^ Å) with tetrahydrofuran (THF) as the eluent at a flow rate of 1 mL/min at 35 °C. However, THF GPC measurements at 35 °C are not recommended due to the poor solubility of PVDF-based polymers and clogging of the columns.

### Tensile Test

The bulk films were tested in tensile mode by using DMA (TA RSA-G2). The film’s thickness was between 100 and 200 μm. The samples were stretched at a constant tensile rate of 0.05 mm/mm·s at room temperature.

## Results and Discussion

**Figure 1:**
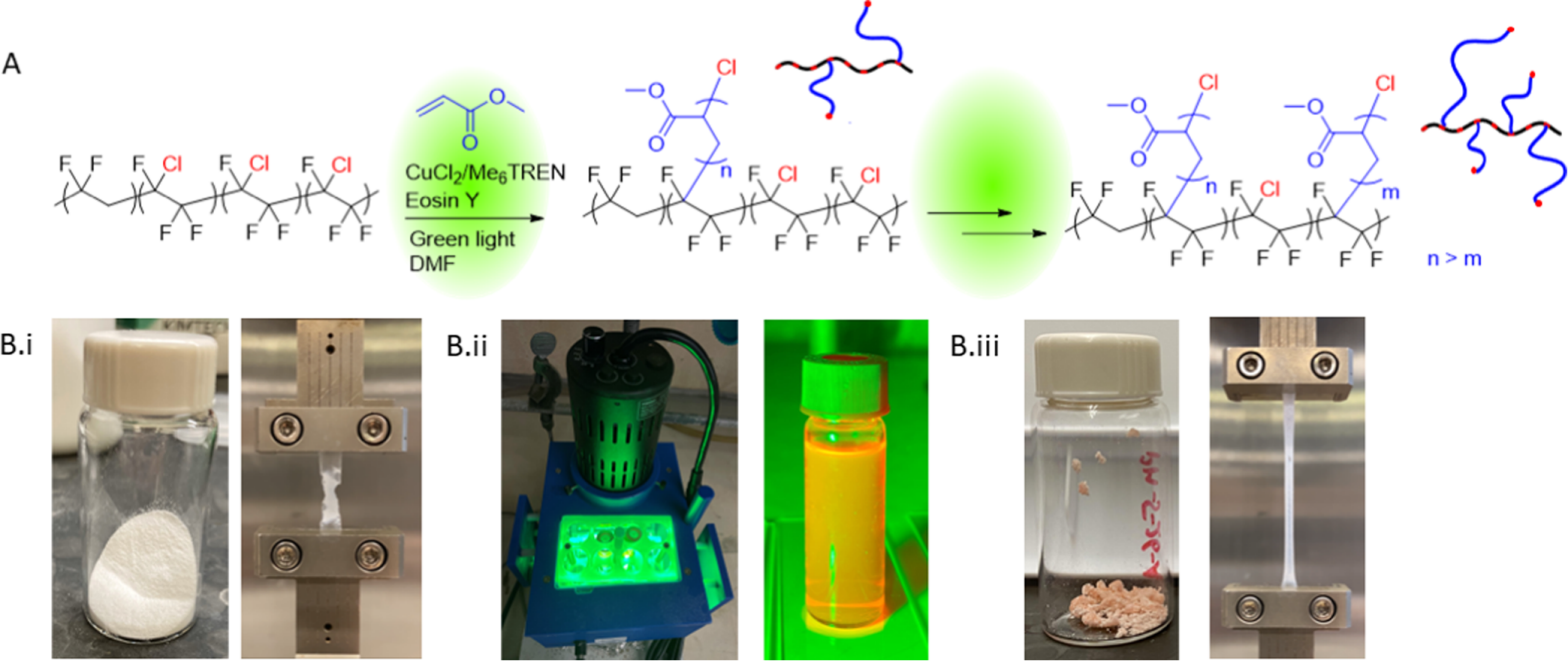
(A) Scheme of eosin Y-mediated grafting from PVDF-CTFE by ATRP together with representative graft architectures at each polymerization time. (B) Modification of PVDF-CTFE: (i) starting PVDF-CTFE material and its representative tensile test at maximum elongation, (ii) grafting reaction, and (iii) grafted PVDF-CTFE and its effect on mechanical properties (example with grafted poly(*n*-butyl acrylate), 21 wt %).

Figure 1A illustrates the grafting of poly(methyl acrylate) from PVDF-CTFE via eosin Y-mediated ATRP together with the anticipated final graft copolymer architectures. PVDF-CTFE features C–Cl activation sites that have lower bond dissociation energies (BDE) and are easier to activate than the C–F sites in pure PVDF. At the same time, it is still more difficult to initiate than typically activated alkyl halides used in ATRP, such as ethyl α-bromoisobutyrate (EBiB), and is a good starting point toward understanding the modification process of PVDF. Figure 1B shows an overview of the modification steps with consequences on the mechanical properties.

## Initial Optimization of the Eosin Y-Mediated ATRP of Methyl Acrylate from PVDF-CTFE

All experiments were done without any deoxygenation in closed 1-dram vials with 0.5–1 mL of air in the headspace. For the study, EvoluChem photoreactors^63^ equipped with a green light source were used (Figure 1B.ii). The polymerization experiments were limited to the two front, and middle positions, as these showed similar exposure to light and provided reproducible monomer conversions. No air cooling was used, as this resulted in uneven temperature gradients across the reactor, i.e., cooler in the proximity of the fan. The arrangement of the lamp source limited the temperature to rise within the photoreactor to 25–29 °C in all our experiments, which reduced the impact on the polymerization kinetics. Stirring (300 rpm, Figure S1A) improved the homogeneity of the viscous polymerization mixtures with a high content of PVDF-CTFE. Light intensity was conveniently adjusted (Figure S1B). Aliquots of the polymerization mixtures were withdrawn, diluted in deuterated acetone, and analyzed by NMR to estimate monomer conversion, which was done according to relative integrals of vinyl proton signals at 5.80–6.44 ppm to methyl ester (OCH_3_) signal at 3.50–3.91 ppm (details in Figure S2).

The initial polymerizations of MA were carried out under the lowest green light intensity (25%, 6.20 ± 0.71 mW/cm^2^) in a mixed DMF/DMSO solvent, 0.116 g/mL concentrations of PVDF-CTFE and eosin Y/copper catalyst ratio of 0.3/1.0. Throughout all optimization experiments, the amount of copper catalyst vs MA and the concentration of MA were fixed to 200 ppm mol and 2 M, respectively. First, the effect of solvent composition on the monomer conversion upon 8 h of irradiation was studied (Table 1, entries 1–4). Note that modification of PVDF-based (co)polymers is limited to several solvents in which they dissolve. Acetone, THF, MEK, and MIBK have low polarity and are known to be latent solvents for PVDF, i.e., they become good solvents upon heating; DMF, *N*-methylpyrrolidone (NMP), and DMSO are polar and good solvents also at room temperature. Polar solvents are also preferred in ATRP for faster polymerization kinetics, as well as oxygen tolerance.^64−67^ Importantly, DMSO is also a known oxygen scavenger, which explains its popularity in photopolymerization studies.^66,68^ Increasing the amount of DMSO from 10 to 20% in the solvent mixture dramatically improved the monomer conversion from 5.1 to 40.0% (entries 1–2). Further increases by 2.5 and 4.5 times resulted in smaller improvements, i.e., to 61.8 and 70.6% conversions, respectively (entries 3–4). At the highest DMSO level (90% in the solvent mixture), reducing the amount of eosin Y by 10 times lowered the conversion only to 46.1% (entry 5), while lowering it 3 times gave optimal performance (77.5%, entry 6). Increasing 4 times the amount of ligand, Me_6_TREN, gave a similar result (74.2%, entry 7). A higher excess of ligand was expected to further improve oxygen tolerance; however, it also led to quicker bleaching of eosin Y.

**Table 1:** Optimization of Eosin Y-Mediated ATRP of MA from PVDF-CTFE^a^

no.	DMSO^b^ (%)	Me_6_TREN (eqv.)	EY·Na_2_ (eqv.)	light intensity at 520 nm (mW/cm^2^)	time (h)	conv.^c^ (%)	Mn_th_ (kDa)	Mn_rel_^d^ (kDa)	*Đ*^d^	GD per chain^e^	DP_th_	DP_NMR_^f^	*I*_eff_^g^
1	10	3	0.3	6.20 ± 0.71	8	5.1	116	86	2.22	0.4	1	91	1.1%
2	20	3	0.3	6.20 ± 0.71	8	40.0	172	126	3.40	0.7	8	667	1.2%
3	50	3	0.3	6.20 ± 0.71	8	61.8	207	174	3.55	8.3	12	143	8.4%
4	90	3	0.3	6.20 ± 0.71	8	70.6	221	311	2.01	16.4	14.1	70	29%
5	90	3	0.03	6.20 ± 0.71	8	46.1	182	316	1.80	15.1	9.2	64	14%
6	90	3	0.1	6.20 ± 0.71	8	77.5	232	282	2.15	N.A.	15.5	N.A.	N.A.
7	90	12	0.1	6.20 ± 0.71	8	74.2	226	235	2.89	13.2	14.8	63	23.5%

a MA/Cl/CuCl_2_/Me_6_TREN/EY·Na_2_ = 5000/250/1/x/x, [MA] = 2 M, [PVDF-CTFE] = 0.116 g/mL, [Cu]/[M] = 200 ppm, 25–29 °C, solvent—DMSO/DMF, light intensity 6.20 ± 0.71 mW/cm^2^ at 520 nm.

b *V*_DMSO_/(*V*_DMSO_ + *V*_DMF_)*100%.

c Conversion was calculated from NMR.

d Number-averaged molecular weight and dipersity determined from DMF GPC with calibration against poly(methyl methacrylate) (PMMA).

e Grafting density determined from NMR.

f PMA degree of polymerization determined from NMR.

g Initiation efficiency determined as DP_th_/DP_NMR_*100%. Calculations were carried out according to NMR spectra given in Figures S7–S12 (details in captions).

## Molecular Characterization

**Figure 2:**
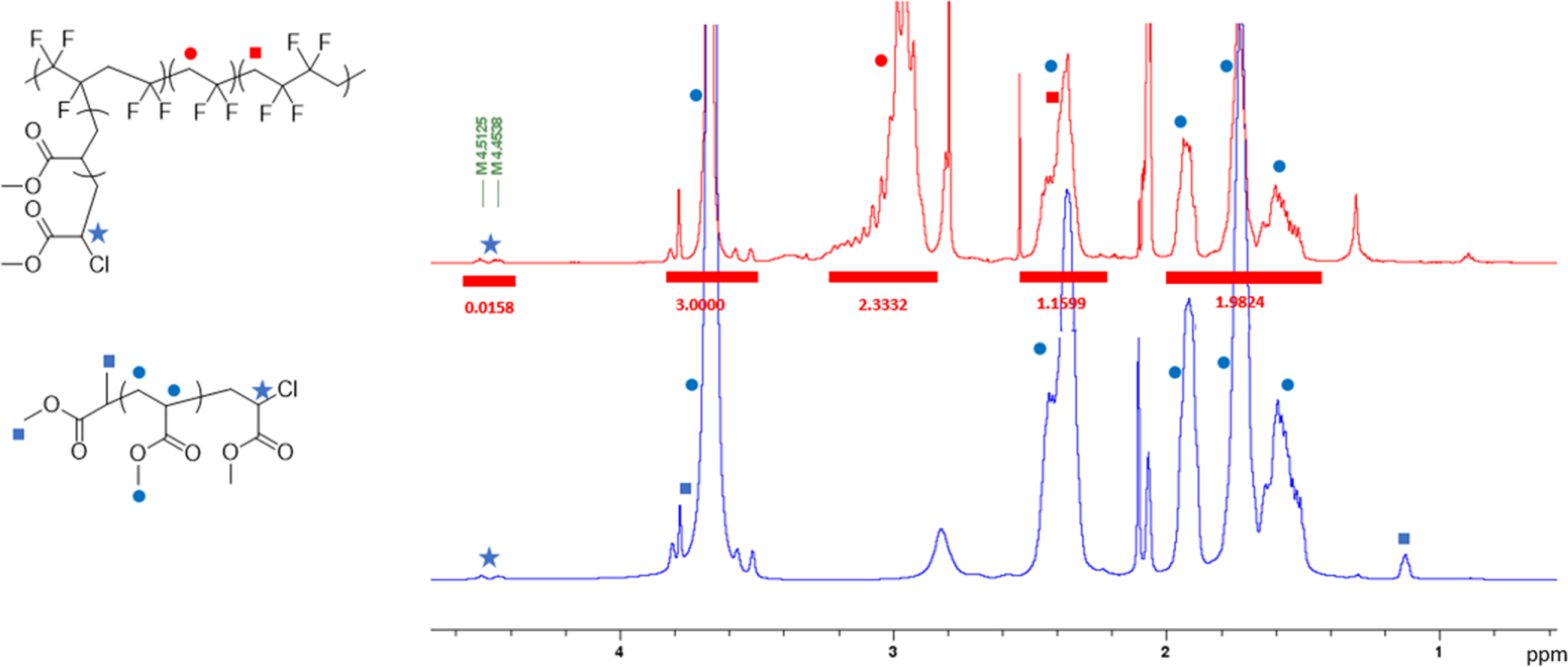
^1^H NMR spectra of (top, red) PVDF-CTFE-*g*-PMA and (bottom, blue) PMA. A signal of C**H**Cl at 4.47 ppm was compared with a C**H**_**2**_CF_2_ signal at 2.9 ppm to determine the grafting density of PMA per PVDF-CTFE chain. A signal of COOC**H**_**3**_ was compared with the C**H**_**2**_CF_2_ signal to determine the PMA degree of polymerization.

The PMA grafts give a unique opportunity to evaluate chain-ends due to their distinct Br–CH methine group. To this end, NMR spectra of purified and dried samples were recorded (256 scans with a cryoprobe for better accuracy) and compared with spectra of pristine PVDF and PVDF-CTFE (Figures S3–S6). The ω-chain-end PMA-Cl signals were clearly visible (Figure 2), which have never been reported before. Their integrals were used to estimate average degree of polymerization of PMA (DP) and grafting densities (GD per chain). It must be emphasized that the grafts on PVDF-based polymers cannot be cleaved and are otherwise very difficult to analyze. Previous approach relied on an additional hydrogenation step to convert unreacted CTFE groups into CF_2_CFH to back-calculate grafting densities according to the NMR signal at 5.3–5.8 ppm.^69^ Alternatively, adjacent groups to the attachment of grafts, e.g., −CF_2_CF(PMMA)**CH**_**2**_CF_2_–, may be observed in F–H decoupled ^1^H NMR spectra and were successfully used to determine GD in a more direct method.^70^ The decoupling is essential to limit the overlap with the backbone signals. Our observation simplifies the analysis. The fact that we observed the chain-ends confirms good control of the polymerization and is an important step toward understanding the grafting of PVDF-based materials. Average degrees of polymerization of PMA and grafting densities are given in Table 1 and are based on calculations below NMR spectra in Figures S7–S12. Typically for a poorly initiating system, the grafting density increased with conversion. Less than one grafted chain below 40% conversion and more than eight above 60% were observed. Maximum grafting density was 16.4 per PVDF-CTFE chain with a 29% initiation efficiency. More DMSO resulted in higher conversions and higher grafting densities and as such may be used to tune the architecture of the grafted copolymers. Polymerizations from PVDF, as a poor initiating system with mechanistically ill-defined process, are plagued by the formation of unattached homopolymers, especially when light-mediated process is used.^23,29,69,71,72^ These require laborious and repeated precipitations into selective solvents (e.g., PMMA/PMA-selective chloroform) for quantitative separation.

Diffusion-ordered NMR spectroscopy (DOSY NMR) was used to confirm the grafting as well as to exclude formation of unattached homopolymers. A DOSY experiment utilizes two gradient sequences, one marks the location of molecules in space, while the other reads out how many molecules remained in their initial location.^73^ This is accomplished by encoding a corkscrew pattern of magnetization arrows. After time Δ, some molecules diffuse into other locations, which causes mixing of the encoded pattern, and upon decoding, only part of the initial magnetization can be recovered. Conventionally the diffusion coefficient is extracted from the signal by fitting a single component to the Stejskal–Tanner equation:^74^
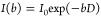
1where *I*_0_ is the signal intensity before decay (*b* = 0), *D* is diffusion coefficient, and
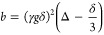
2where γ is the magnetogyric ratio, *g* is the gradient strength, and δ is the gradient pulse duration.

However, such a monoexponential model needs to be corrected in the case of polydisperse samples (see Figures S13, S14). For such system, the proper way would be to either apply inverse laplace transform (ILT) algorithm tuned for broad diffusion distribution^75,76^ or to fit a more adequate model of the signal. In this work, we utilized the gamma distribution model,^77^ where the relation between the signal and diffusion coefficients is described as
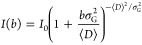
3where σ_G_ is the standard deviation in the gamma distribution model and ⟨*D*⟩ is the mean diffusion coefficient.

**Figure 3:**
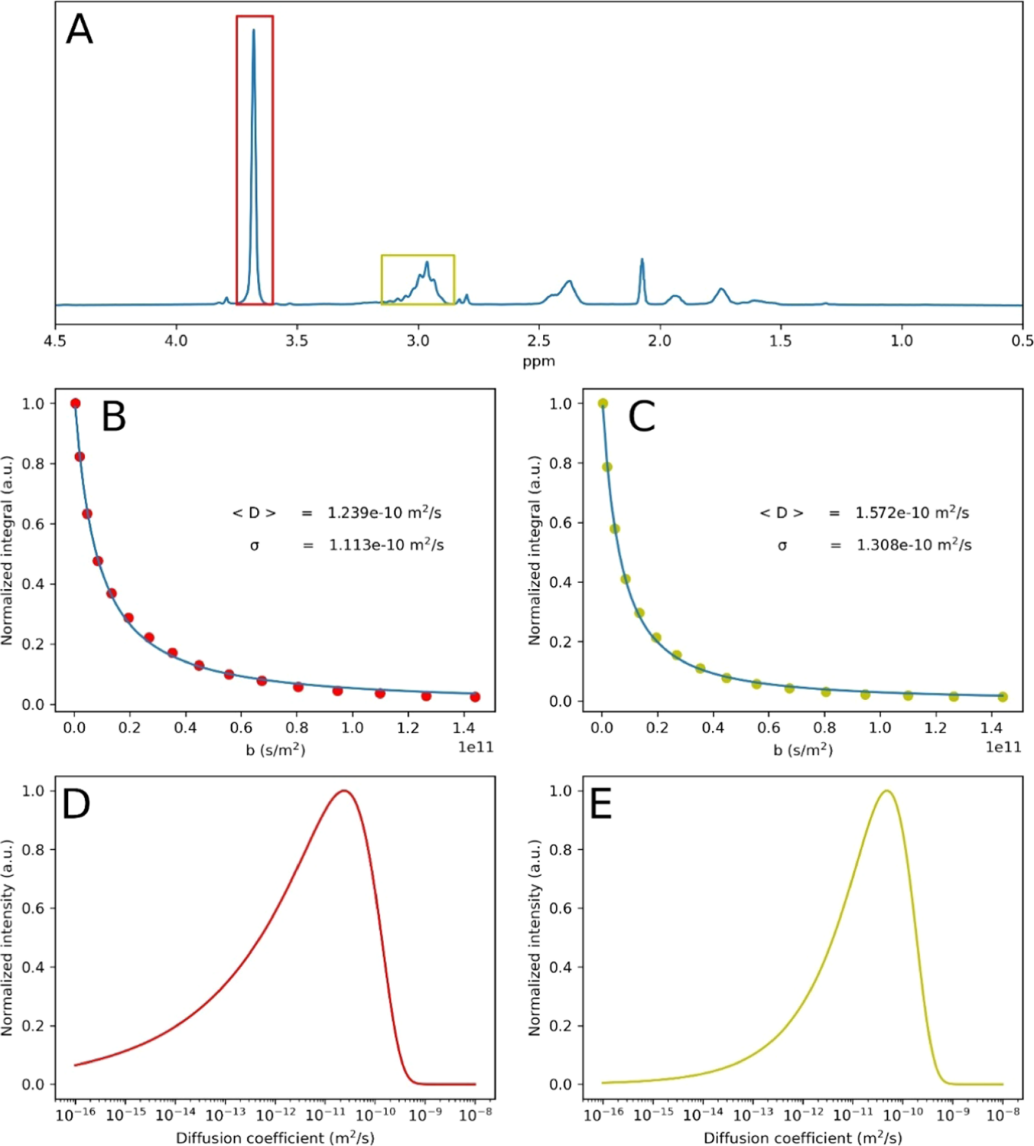
Comparison of diffusion coefficients of the components of PVDF-CTFE-*g*-PMA; the grafts (OCH_3_ signal at 3.6 ppm, red) and the backbone (CH_2_–CF_2_ signal at 2.98 ppm, yellow). (A) 1D ^1^H NMR spectrum of the sample. (B, C) Quality fits of the gamma-model for the OCH_3_ and CH_2_–CF_2_. (D, E) Diffusion coefficient distributions of the peaks. DOSY NMR was carried out in acetone-*d*_6_.

The fits and diffusion distribution for the methoxy signal of PMA at 3.67 ppm and peak at 2.98 ppm for CH_2_CF_2_ of PVDF-CTFE are shown in Figure 3. For both peaks, the average diffusion coefficient and the standard deviation are in good agreement. The σ_G_ value suggests a broad dispersity of the polymer.

## Grafting under High Light Intensities

The next step was to maximize the monomer conversion in 1 h, i.e., 8 times quicker than before. While within this time frame, the previous conditions (Table 1, entry 7) resulted in only 13.8% conversion of MA (Table 2, entry 2), incrementally doubling the light intensity to 50% (11.8 ± 1.5 mW/cm^2^) and 100% intensity (23.8 ± 2.0 mW/cm^2^) enabled conversions of MA to 44.0% (entry 4) and 73.8% (entry 6). The conversion at the highest intensity after 1 h matches the result obtained at 4 times lower light intensity after 8 h. Strikingly, the same reaction without PVDF-CTFE initiator gave negligible conversion of MA (<1%, control), in accordance with the DOSY NMR experiment above. This observation points toward a very limited (if any) generation of unattached homopolymers, and the results strongly suggest high selectivity of the grafting method.

**Table 2:** Effect of Light Intensity on Conversion of MA during Eosin Y-Mediated ATRP from PVDF-CTFE^a^

no.	light intensity at 520 nm (mW/cm^2^)	temperature (°C)	time (h)	conv.^b^ (%)	Mn_th_ (kDa)	Mn_rel_^c^ (kDa)	*Đ*^c^	GD per chain^d^	DP_th_	DP_NMR_^e^	*I*_eff_^f^
1	6.20 ± 0.71	25.6	0.6	7.9	121	N.A.	N.A.	N.A.	1.6	N.A.	N.A.
2	6.20 ± 0.71	25.6	1	13.8	130	83.4	2.40	5.3	2.8	49	5.7%
3	11.8 ± 1.5	26	0.6	19.3	139	N.A.	N.A.	N.A.	3.9	N.A.	N.A.
4	11.8 ± 1.5	26	1	44.0	178	200	2.70	11	8.8	63	14.0%
5	23.8 ± 2.0	28.2	0.6	53.4	193	283	1.87	N.A.	10.7	N.A.	N.A.
6	23.8 ± 2.0	28.2	1	73.8	226	266	2.16	15.6	14.8	85	17.4%
control	23.8 ± 2.0	28.2	1	<1	N.A.	N.A.	N.A.	N.A.	N.A.	N.A.	N.A.

a MA/Cl/CuCl_2_/Me_6_TREN/EY·Na_2_ = 5000/250/1/12/0.1, [MA] = 2 M, [PVDF-CTFE] = 0.116 g/mL, [Cu]/[M] = 200 ppm, DMSO/DMF = 8/2, v/v.

b Conversion determined from NMR.

c Number-averaged molecular weight and dipersity determined from DMF GPC with calibration against PMMA.

d Grafting density determined from NMR.

e PMA degree of polymerization determined from NMR.

f Initiation efficiency determined as DP_th_/DP_NMR_*100%, control was carried out without PVDF-CTFE. Calculations were carried out according to NMR spectra given in Figures S15–S17 (details in captions).

## Kinetic Studies

**Figure 4:**
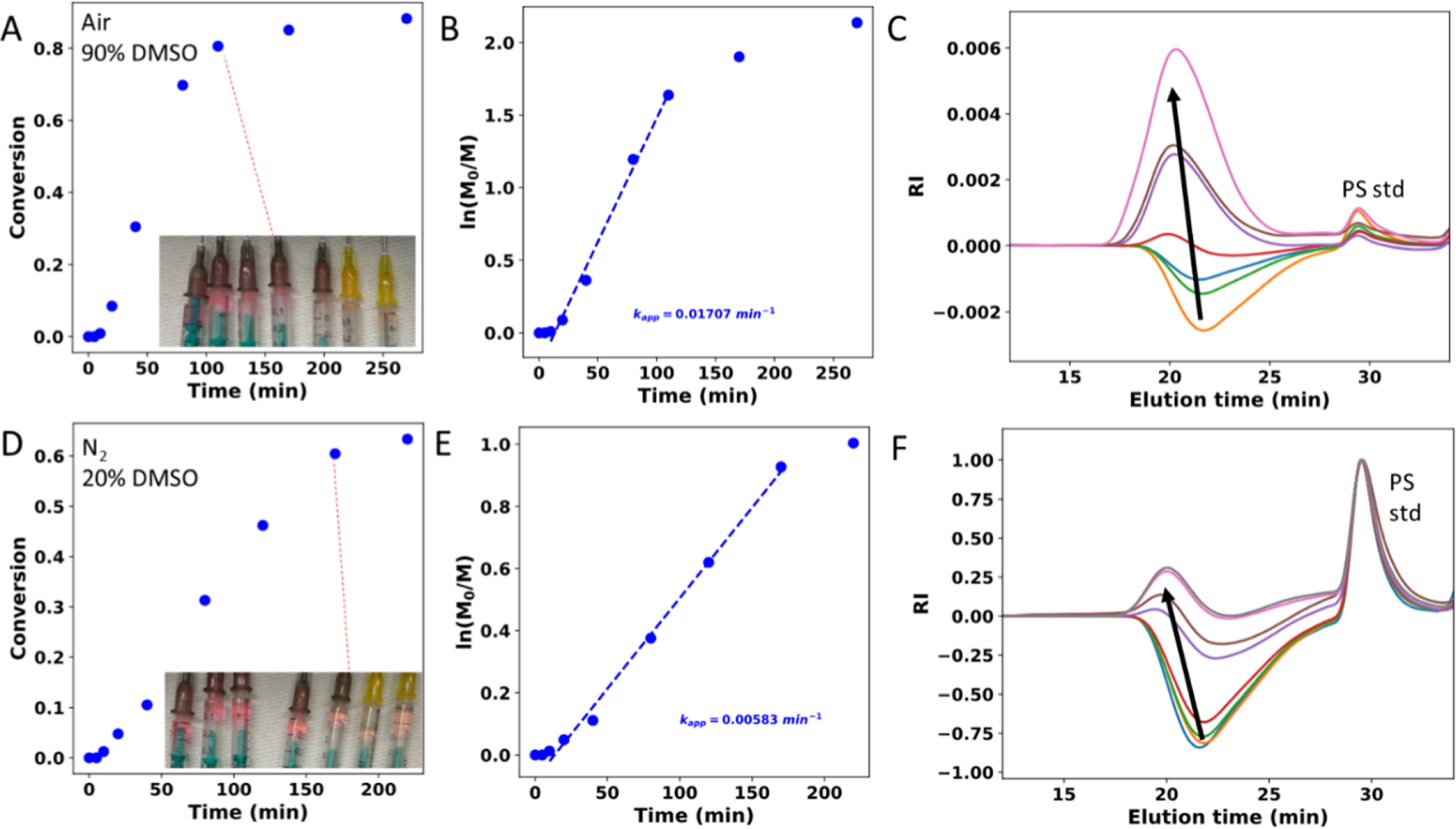
Effect of solvent composition on oxygen tolerance and kinetics of the polymerization of MA from PVDF-CTFE; (A–C) *V*_DMSO_/(*V*_DMSO_ + *V*_DMF_)*100% = 90%, (D–F) *V*_DMSO_/(*V*_DMSO_ + *V*_DMF_)*100% = 90 or 20%. Conversion of MA is limited by bleaching of eosin Y (insets in (A) and (D)) and occurs at 2 h (A) and 3 h (D). Conversion of the monomer was monitored by NMR. Molecular weight was monitored by DMF GPC. Conditions: MA/Cl/CuCl_2_/Me_6_TREN/EY·Na_2_ = 5000/250/1/3/0.1, 23.8 ± 2.0 mW/cm^2^ at 520 nm.

Two solvent systems for grafting monomers of high and low polarity were tested, i.e., using 1/9 and 8/2 DMF/DMSO, v/v, respectively. While MA was used in both experiments for direct kinetic comparison, these compositions are compatible with other monomers, such as poly(ethylene glycol) methyl ether acrylate and *n*-butyl acrylate (see the Conclusions section). The presence of DMSO is important for oxygen tolerance and an easy experimental setup. Note that in these experiments the excess of the Me_6_TREN ligand was reduced 4 times, as otherwise fast bleaching of eosin Y and limited conversions were observed. Figure 4A,B,D,E shows the progress of the polymerizations of MA from PVDF-CTFE. The polymerizations leveled off at 120 min for 90% DMSO and 180 min for 20% DMSO. This moment coincided with the onset of bleaching of eosin Y (insets in Figure 4A,D). The polymerization with 90% DMSO was nearly 3 times faster than the one with 20% DMSO (*k*_p,app_ = 0.0107 vs 0.00583 min^–1^). The polymerization with 20% DMSO displayed only limited oxygen tolerance, and taking aliquots under air was challenging. Therefore, it was carried out under nitrogen. Figure 4C,F shows the evolution of the PVDF-CTFE-*g*-PMA signal in DMF GPC at different polymerization times. A small shift toward lower elution volumes (higher molecular weights) was observed. At the same time, the RI of the signal became less negative and finally more positive upon incorporation of PMA grafts. The negative RI signal is a consequence of lower refractive index of the polymer (*n*_PVDF_ = 1.426) than the DMF eluent (*n*_DMF_ = 1.431). Because of this large refractive index–composition dependence, evaluation of absolute molecular weights by MALS was unreliable. While THF GPC gave positive and more visible signals (Figure S18), due to better refractive index contrast (*n*_THF_ = 1.407), we often observed clogging of the GPC columns and refrained from these analyses. THF is in fact a latent solvent for PVDF and GPC analysis; low temperatures (e.g., 30 °C) should be avoided.

## Modification of Other PVDF-Based Materials

**Figure 5:**
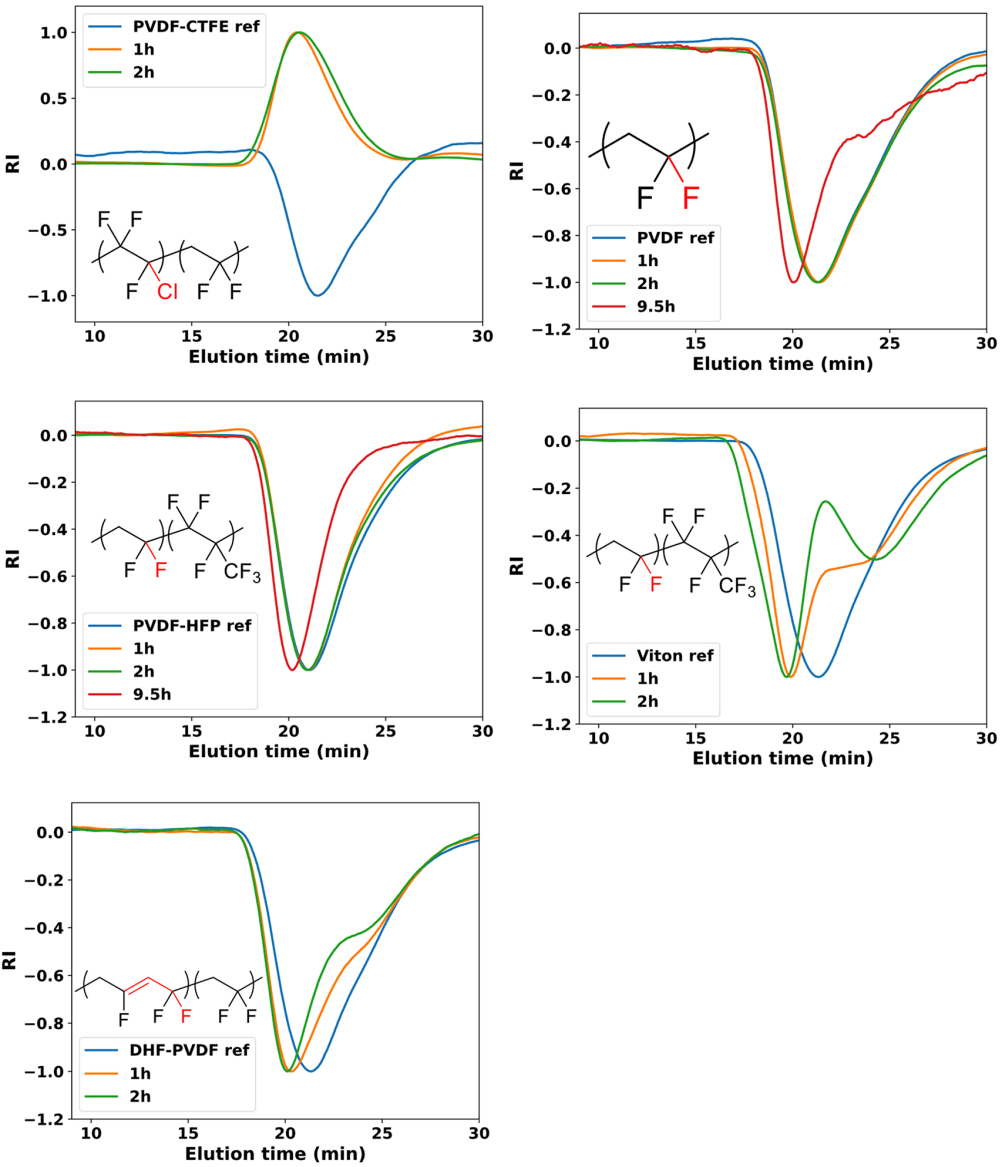
Chemical structures of the PVDF-based polymers used for the grafting of PMA together with GPC traces obtained by DMF GPC.

**Figure 6:**
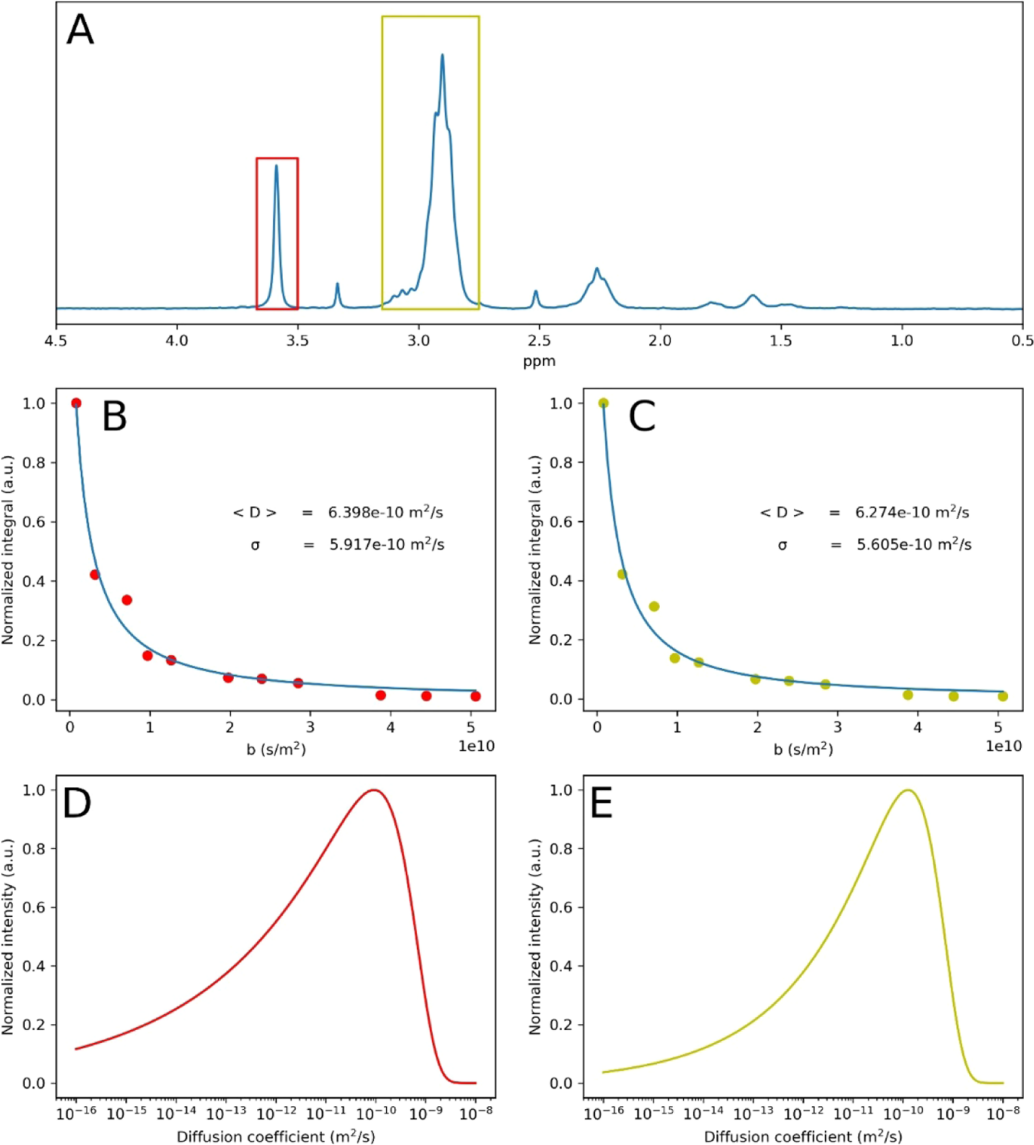
Comparison of diffusion coefficients of the components of PVDF-*g*-PMA; the grafts (OCH_3_ signal at 3.6 ppm, red) and the backbone (CH_2_–CF_2_ signal at 2.98 ppm, yellow). (A) 1D ^1^H NMR spectrum of the sample. (B, C) Quality fits of the gamma-model for the OCH_3_ and CH_2_–CF_2_. (D, E) Diffusion coefficient distributions of the peaks. DOSY NMR was carried out in DMSO-*d*_6_.

Finally, we investigated whether the eosin Y-mediated ATRP can be used for grafting from more challenging PVDF-based (co)polymers, i.e., polymers uniquely with strong C–F bonds, viz., PVDF, PVDF-HFP (poly(vinylidene fluoride-*co*-hexafluoropropylene)), DHF-PVDF (dehydrofluorinated PVDF), and a representative fluorocarbon elastomer, Viton A401C. PVDF-HFP-based fluorocarbon elastomers (FKMs) comprise at least 20 mol % HFP and, since their commercialization in 1957, have gained popularity owing to great mechanical performance, thermal stability (service temperature of 200 °C), chemical inertness, and low swelling in oil and petroleum. Their operation in hostile environments is further improved by cross-linking.^78,79^ They are often used in gaskets, O-rings, and diaphragms for automotive, aerospace, and oil industries. Eosin Y-mediated ATRP resulted in minimal conversion and change in GPC trace after 2 h modification of PVDF (4.3%, Table 3, entry 4) and PVDF-HFP (2.8%, entry 7), which indicates high selectivity of the method toward activation of C–Cl vs C–F bonds. Interestingly, however, extending the polymerization time to 9.5 h converted ca. 22% of MA (entry 9), and more importantly, the GPC trace of this sample clearly shifted toward lower elution volumes (higher molecular weights), which confirmed successful grafting (Figure 5). Another proof for successful and selective grafting was provided by DOSY NMR. Similarly to the grafting from PVDF-CTFE, the obtained diffusion values for methoxy signal of PMA at 3.67 ppm and a peak at 2.98 ppm for CH_2_CF_2_ were in good agreement (Figure 6). These values clearly decreased, as compared to the unmodified PVDF (Figure S19), i.e., ⟨*D*⟩ decreased from 1.8 × 10^–9^ to 6.3–6.4 × 10^–10^ m^2^ s^–1^, while σ_G_ decreased from 1.6 × 10^–9^ to 5.6–5.9 × 10^–10^ m^2^ s^–1^, which indicates that molecular weight increased, while molecular weight distribution decreased upon grafting.

**Table 3:** Comparison of the Eosin Y-Mediated ATRP with Other PVDF-Based Polymers^a^

no	PVDF material	time (h)	conv. (%)	initial Mn_rel_ (kDa)^b^	*Đ*^b^	final Mn_rel_ (kDa)^b^	*Đ*^b^	φ_graft_ (wt %)
1	PVDF-CTFE	1	57.5	128	2.00	221	1.87	45.8
2	PVDF-CTFE	2	79.3	128	2.00	195	2.12	53.8
3	PVDF	1	1.1	81	3.48	94	2.54	1.6
4	PVDF	2	4.3	81	3.48	95	2.64	5.9
5	PVDF	9.5	21.6	81	3.48	131	3.08	24.1
6	PVDF-HFP	1	0.4	115	2.72	140	2.13	0.6
7	PVDF-HFP	2	2.8	115	2.72	114	2.48	4.0
8	PVDF-HFP	9.5	22.7	115	2.72	265	1.84	25.0
9	DHF-PVDF	1	21.3	86	3.86	93	3.60	23.9
10	DHF-PVDF	2	21.4	86	3.86	91	4.12	23.9
11	Viton A401C	1	21	123	2.89	65	7.60	23.6
12	Viton A401C	2	26	123	2.89	76	10.40	27.7

a Conditions: MA/Cl/CuCl_2_/Me_6_TREN/EY·Na_2_ = 5000/250/1/12/0.1, *V*_DMSO_/(*V*_DMSO_ + *V*_DMF_)*100% = 90%. 23.8 ± 2.0 mW/cm^2^.

b Number-averaged molecular weight and dipersity determined from DMF GPC with calibration against PMMA; PVDF-HFP, poly(vinylidene fluoride-*co*-hexafluoropropylene); DHF-PVDF, dehydrofluorinated (9 mol %) poly(vinylidene fluoride).

What is striking and is in sharp contrast to grafting from PVDF-CTFE is that the initiation was clearly delayed, which suggests preactivation in the initial reaction period. The precise mechanism is unclear. Some authors suggested easier initiation from C–F at HH defects.^42^ More likely possibility is an exchange of fluorine by chlorine atoms prior to the polymerization forming more active C–Cl sites, as proposed by the group of Chatterjee, who observed C–Cl signatures in X-ray photoelectron spectroscopy (XPS) scans.^80^ Another mechanism involves unsaturations, whose importance during grafting was raised in a perspective paper by Ladmiral.^40^ In this scenario, the polymerization happens via a grafting-through mechanism. In our grafting experiments, unsaturations clearly did play an important role. Prolonged exposure to Cu/Me_6_TREN led to dehydrochlorination of PVDF-CTFE, as marked by vinyl proton signals in ^1^H NMR spectra of the grafted PVDF-CTFE-*g*-PMA (an experiment with 12 equiv of Me_6_TREN) (Figure S20). Similarly, dehydrofluorination of PVDF is likely to occur, albeit at a slower rate. These unsaturations may also activate nearby C–F bonds, similarly as in the case of allyl halide initiators (chlorides and halides).^43^ These vinyl groups, however, were difficult to observe on the grafted PVDF. Thus, we performed control reactions mimicking the polymerization conditions, but without the monomer that would otherwise react with the vinyl groups. These experiments indeed indicated dehydrofluorination of PVDF in the presence of ATRP ligands (NMR spectra in Figure S21). The vinyl proton signal was observed at 7.23 ppm, and interestingly it became larger when more Me_6_TREN was used. This is the first time the dehydrofluorination during ATRP was observed. Besides NMR, dehydrofluorination of PVDF may also be followed by combustion elemental analysis^81^ or X-ray photoelectron spectroscopy (XPS),^81,82^ as progressing dehydrofluorination leads to smaller fluorine content, as well as a distinct C=C signal at C 1s high-resolution XPS scan. However, previous attempts to detect unsaturations after ATRP-mediated grafting were unsuccessful,^80^ which may be due to detection challenges of extremely small content of these unsaturations.

In view of these findings, grafting from PVDF-DHF is particularly interesting. As expected, initiation was quick, and the monomer conversion reached 21.3% already within the first hour. At the same time, GPC signal clearly shifted toward lower elution volumes (Figure 5). Similar activation was observed for Viton A401C (Table 3, entry 11, Figure 5), which contains additives, bisphenol AF, and benzyltriphenylphosphonium salt, acting as cross-linkers. The plausible mechanism involves HF elimination and substitution of the bisphenol onto the formed double bonds.^83^ While this cross-linking process typically proceeds at higher temperatures above 160 °C, it may be accelerated in the presence of amines. It is clear that these additives helped to initiate grafting during ATRP, but they may also have led to cross-linking. This was marked by drastically increased dispersities; from initial 2.89 to 7.60 (entry 11) and 10.40 (entry 12) after 1 and 2 h of the grafting, respectively.

## Preparation of Graft Copolymers with Other (Meth)acrylate Monomers

The eosin Y-mediated ATRP from PVDF-based materials was applicable to a range of (meth)acrylate monomers. We successfully grafted poly(*tert*-butyl acrylate) (PtBA) from PVDF-CTFE in anisole reaching ca. 50% conversions of tBA within 80 min using aspectLED light source (details in the Supporting Information, *T* = 60 °C) as listed in Table S1 (kinetic plots in Figure S22 and GPC traces in Figure S23). Similarly, poly(ethylene glycol) methyl ether acrylate (PEGMEA, average *M*_n_ = 480) and lauryl acrylate were grafted from PVDF-CTFE in DMF (Table S2). Materials with mixed compositions of PEGMEA, tBA, and 2-cyanoethyl acrylate (CEA) were also successfully obtained (Table S3).

**Figure 7:**
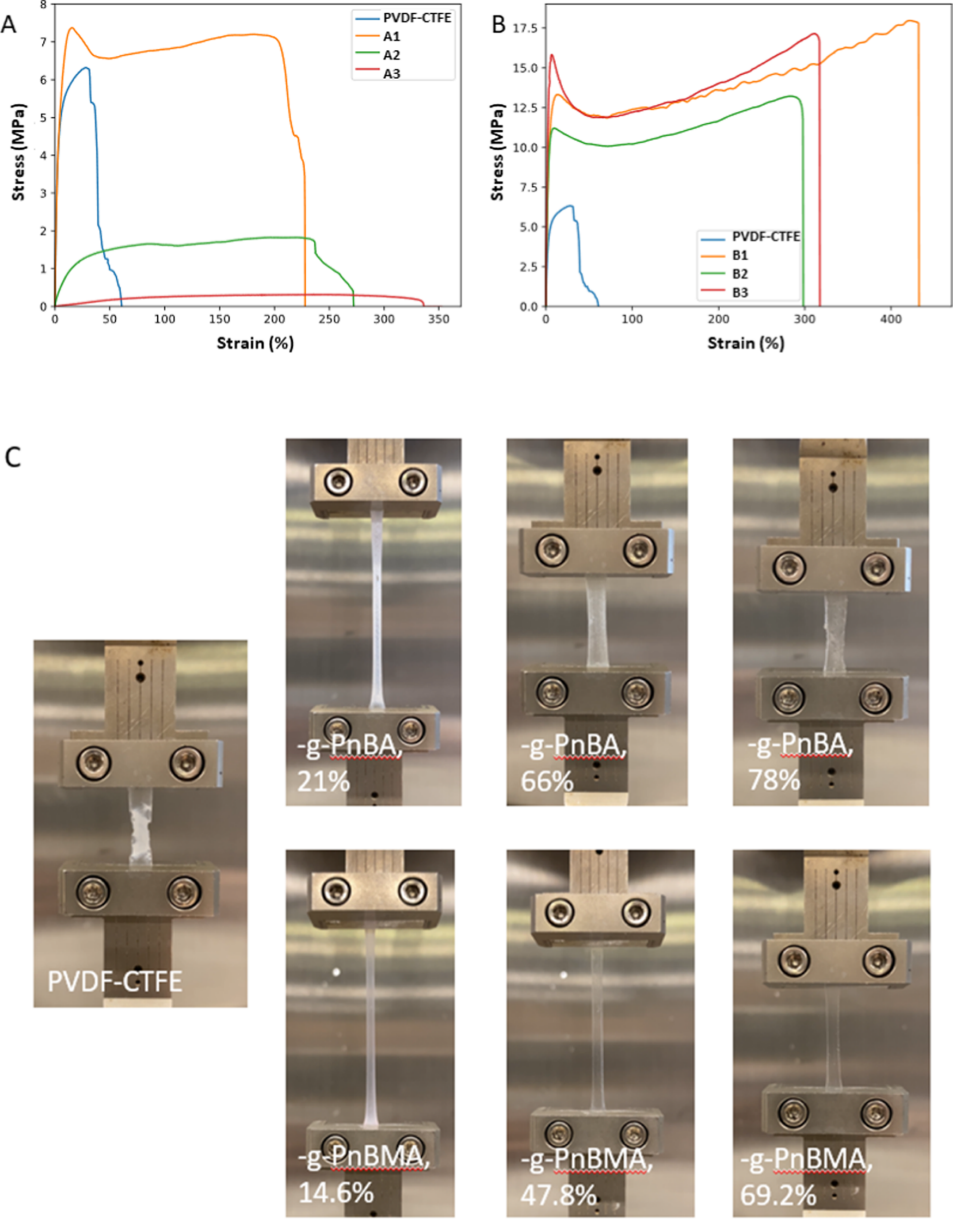
Tensile tests of PVDF-CTFE grafted with PnBA (A) and PnBMA (B). Snapshots of the tensile tests at maximum elongations (C). Videos S1 and S2, Supporting Information.

Poly(*n*-butyl acrylate) (PnBA) and poly(*n*-butyl methacrylate) (PnBMA) were grafted from PVDF-CTFE to prepare materials of higher ductility, as these grafts have low glass transition temperatures (*T*_g,PnBA_ = −53 °C, *T*_g,PnBMA_ = 20 °C) and dilute crystalline domains of PVDF. As BA and BMA are hydrophobic monomers, the ratio of solvents DMSO/DMF of 2:8 was used to prevent early precipitation of the grafted copolymers. A set of samples with varied contents of PnBA (21–78 wt %) and PnBMA (15–69 wt %) were obtained (Table 4). FTIR spectra examples of similar grafts are given in Figures S24 and S25. Upon grafting, clear changes are observed including C–H stretching bands at 2962/2959, 2936, and 2875 cm^–1^ that overshadow small signals of PVDF-CTFE at 3025 and 2982 cm^–1^, as well as strong carbonyl stretching band at 1734/1728 cm^–1^. The crystallinity of the samples, as determined by DSC (Figures S26 and S27), decreased, as expected upon the grafting. When calculated per PVDF fraction, crystallinity remained relatively high, i.e., 6–18%, as compared to 24.3% of the unmodified PVDF-CTFE. Melting and crystallization temperatures decreased slightly for PnBA grafts, i.e., by 7.4 and 24 °C, respectively. This is a usual case for blends and copolymers of crystalline and amorphous components and is due to thermodynamic melting point depression.^84^ Surprisingly, however, the opposite trend was observed for PnBMA grafts. For a sample with 14.6 wt % of PnBMA, melting and crystallization temperatures increased by 2.2 and 5.2 °C, respectively. A sample with more PnBMA, i.e., 47.8 wt %, showed additional crystallization transitions at 68.1 (depression by 50.3 °C). This behavior must be due to other factors such as morphology changes upon grafting or increased tension of the backbone exerted by the grafted chains.^85^ Two crystallization transitions occurring for the 47.8 wt % sample may also indicate the presence of two grafted populations of strikingly different behavior, i.e., loosely and densely grafted PVDF-CTFE. These samples were subsequently solvent cast from DMF solutions into Teflon molds. After drying, they were tested on a dynamic mechanical analyzer in tensile mode, as shown by representative stress–strain curves in Figure 7 (Videos S1 and S2, Supporting Information). The grafting indeed increased ductility (up to 390% from 35.5% for PnBA grafts) and drastically improved toughness (up to 46 × 10^6^ from 1.8 × 10^6^ J/m^3^ for PnBMA grafts). PnBMA grafts did not deteriorate Young’s moduli, and PnBA grafts gradually softened PVDF-CTFE with increasing graft content (Table 5). A literature example with PVDF grafted with PnBMA by conventional ATRP gave materials of similar ductilities (up to 422 from 16.5%), as well as stresses at break (9.6–15.2 MPa).^86^ However, the ductility improved 25.6 times for PVDF, while it improved only 11 times for PVDF-CTFE. These two behaviors are well mirrored by crystallinity changes of the two materials. The grafted PVDF-CTFE was only slightly less crystalline than the grafted PVDF (3.7–15 vs 10–21%). However, the crystallinities of the starting materials were much different, i.e., 24.3% for PVDF-CTFE vs 52% for PVDF. Mechanically very promising are also grafts of PDMAEMA (PVDF-*g*-PDMAEMA), which were reported with 45 times improved ductility (750% elongation at break) and 20 times higher toughness for a sample with 40 wt % of PDMAEMA grafts.^42^ We recently reported PVDF graft copolymer binders for battery applications. PVDF-CTFE-*g*-P(PEGMEA-*co*-AA) improved elongation at break from 92.1 to 319% and toughness from 10 to 23.7 mJ/m^3^, only upon grafting of 20 wt % of the polyacrylates.^87^ These excellent mechanical improvements were likely caused by beneficial hydrogen bonding interactions.

**Table 4:** Polymerization Results for Grafting PnBA and PnBMA from PVDF-CTFE (10 wt % of CTFE)^a^

nBA						
DP_t_	monomer/PVDF-CTFE w/w	polymerization time (h)	conv.	φ(graft), wt %	χ(PVDF)	χ
20	2.2/1	0.5	11.8%	21%	6.2%	4.9%
50	5.5/1	1.5	35%	66%	17.7%	6.0%
200	22.0/1	0.5	16.4%	78%	8.3%	1.8%

nBMA						
DP_t_	monomer/PVDF-CTFE w/w	polymerization time (h)	conv.	φ(graft), wt %	χ(PVDF)	χ
20	2.4/1	1.33	7.0%	14.6%	17.6%	15.0%
50	6.1/1	1.33	15.0%	47.8%	9.6%	5.0%
200	24.4/1	1.33	9.2%	69.2%	11.9%	3.7%

a Crystallinity values were obtained from DSC (Figures S26, S27).

**Table 5:** Mechanical Properties of PVDF-CTFE and PVDF-CTFE Grafted with PnBA and PnBMA

sample	φ(graft), wt %	Young’s modulus (MPa)	toughness (10^6^ J/m^3^)	elongation at break (%)
PVDF-CTFE	0.0%	234.2 ± 5.4	1.8 ± 0.7	35.5 ± 4.5
PVDF-CTFE-*g*-PnBA				
A1	21.0%	298 ± 47	26.0 ± 5.4	253 ± 39
A2	66.0%	18.5 ± 1.8	3.1 ± 0.9	199 ± 36
A3	78.0%	1.4 ± 1.2	1.1 ± 0.6	390 ± 170
PVDF-CTFE-*g*-PnBMA				
B1	14.6%	488 ± 47	46 ± 14	316 ± 86
B2	47.8%	445 ± 45	22 ± 12	210 ± 100
B3	69.2%	460 ± 150	24 ± 16	211 ± 76

## Conclusions

This manuscript presents a facile, oxygen-tolerant, and fast (1 h) synthetic method to modify PVDF-based materials using inexpensive, accessible eosin Y as a mediator of ATRP under green light irradiation. The process was conducted using solvents of low and high polarity (anisole, DMF/DMSO) making it applicable to a wide range of monomers such as low polarity lauryl acrylate, *tert*-butyl acrylate, or *n*-butyl (meth)acrylate, as well as more polar 2-cyanoethyl acrylate and poly(ethylene glycol) methyl ether acrylate. The successful grafting resulted in an increase in molecular weight, as indicated by lower elution volumes in GPC, as well as lower diffusion coefficients in DOSY NMR experiments. The latter also unambiguously excluded the formation of unattached homopolymers. Chain-end analysis of PVDF-CTFE-*g*-PMA materials indicated that grafting density, as well as length of the grafts could be easily tuned with solvent composition, as well as light intensity, with maximum PMA DP of 85 and GD of 15.6 using 90% DMSO and 23.8 mW/cm^2^ light intensity. Nonchlorinated PVDF-based materials required longer activation periods (9.5 h vs 1 h) for the grafting to reach substantial conversions (20% of MA). It was demonstrated by control reactions that the ATRP ligands facilitate the formation of unsaturations within the PVDF backbone, which likely activate initiating sites. Dehydrofluorinated PVDF, as well as the commercial fluorocarbon elastomer Viton A401C, were 20 times more active than saturated PVDF.

A range of grafted polymer materials were prepared, out of which PVDF-CTFE-*g*-PnB(M)A was carefully studied. The crystallinity of PVDF was not significantly affected (decrease from 24.3 to 12–18%), and the grafting had only a rather diluting effect. In turn, the prepared samples maintained high Young’s moduli, while the ductility and toughness were greatly improved. The presented grafting method may be used for a facile generation of a wide range of grafted copolymer PVDF-based materials, where the original properties of PVDF are not compromised. The method has the potential to readily generate advanced materials with potential use as cathode binders for lithium-ion batteries as well as piezoelectric power generators, actuators, and separation membranes.

## Supporting Information Available

Tensile test (Video S1) (MP4)
Tensile test (Video S2) (MP4)
Polymerization conditions and conversions for ATRP of other monomers (tBA, LA, PEGMEA480, CEA) and the molecular weight characterization of the obtained polymers; images of photochemical reactors; NMR spectra of reaction aliquots; ^19^F NMR, ^1^H NMR spectra of PVDF, PVDF-CTFE; ^1^H NMR spectra of dry PVDF-CTFE-*g*-PMA samples; fittings of DOSY NMR data; DOSY NMR of PVDF-CTFE, PVDF, PVDF-CTFE-*g*-PMA, and PVDF-*g*-PMA; GPC traces of PVDF-CTFE-*g*-PMA in THF eluent; DSC of PVDF-CTFE-*g*-PnBA and PVDF-CTFE-*g*-PnBMA; and additional data for GPC analysis of PVDF-CTFE-*g*-PMA in THF eluent (PDF)
