# Supplementary material for: Selective and Controlled Grafting from PVDF-Based Materials by Oxygen-Tolerant Green-Light-Mediated ATRP
Source: ACS Appl Mater Interfaces. 2024 Apr 23;16(18):23932–47. doi: 10.1021/acsami.4c03369 (PMC11082848; doi:10.1021/acsami.4c03369)
Supplement: Supplementary file 3 — am4c03369_si_003.pdf [file am4c03369_si_003.pdf]

Supporting information

Selective and controlled grafting from PVDF-based materials by oxygen-tolerant green-light-mediated ATRP

Piotr Mocny,[a,b] Ting-Chih Lin,[a] Rohan Parekh,[c] Yuqi Zhao,[c] Marek Czarnota[e], Mateusz Urbańczyk[e], Carmel Majidi,[d] Krzysztof Matyjaszewski[, a]*

[a] Department of Chemistry, Carnegie Mellon University, 4400 Fifth Ave. Pittsburgh, PA, 15213, United States

[b] Faculty of Chemistry, University of Warsaw, Pasteura 1, 02-093 Warsaw, Poland

[c] Department of Materials Science & Engineering, Carnegie Mellon University, 5000 Forbes Ave. Pittsburgh, PA 15213, United States

[d] Department of Mechanical Engineering, Carnegie Mellon University, 5000 Forbes Ave. Pittsburgh, PA, 15213, United States

[e] Institute of Physical Chemistry, Polish Academy of Sciences, Kasprzaka 44/52, 01-224 Warsaw, Poland

* Corresponding author: Krzysztof Matyjaszewski km3b@andrew.cmu.edu

Table S1. Molecular weights of PVDF-co-CTFE and obtained (PVDF-co-CTFE)-g-PtBA samples together with polymerization conditions.

$tBA/Cl/CuCl_2/Me_6TREN$/Eosin Y	[M] (M)	Time (min)	Conv. (%)	DP_{th}	$M_{n,th}$	M_n (g/mol, MALS)	dn/dc	Đ
**PVDF-CTFE**	N.A.	N.A.	N.A.	N.A.	N.A.	108,000	-0.021 (DMF)	2.70
**20/1/0.004/0.012/0.0004**	1.67	80	57	11	243,000	420,000	0.0234 (THF)	1.74
**40/1/0.008/0.024/0.0008**	2.03	60	47	19	327,000	370,000	0.0259 (THF)	1.64
**80/1/0.016/0.048/0.0016**	2.59	60	48	38	566,000	323,000	0.0429 (THF)	1.96

Table S2. An overview of experimental procedures for polymerization of different monomers from PVDF-co-CTFE together with the grafting outcomes.

Procedure	Monomer	$[M]_0$ (M)	[M]/ [Cl]	Time (min)	Conv%	wt% graft	Theoretical Mn (g/mol)	dn/dc	Mn (MALS) (g/mol)	Đ
PEG-c	PEGMEA480	0.454	5	140	11.5	19.1	133,600	0.0050	280,000	2.64
PEG-b	PEGMEA480	0.294	2.5	300	16.5	14.5	126,400	N.A.	N.A.	N.A.
	PEGMEA480	1.07	20	100	50	73	649,400	N.A.	N.A.	N.A.
PEG-a	PEGMEA480	1.270	40	100	49.5	89.1	989,300	0.021	646,000	1.43
LA	LA	1.34	40	60	31	76.8	381,200	N.A.	307,000	2.51

In each case 200 ppm Cu and 20 ppm eosin Y relative to the total concentration of monomers were used; in procedures "LA" 400 ppm and 40 ppm eosin Y were used; conversion % was determined using H-NMR spectroscopy using DMSO-d6 solvent. Dispersity for the reactions PEG-a, PEG-b and PEG-c were determined by using GPC with DMF as eluent, while the reaction "LA" was determined by GPC with THF as eluent.

Table S3. An overview of experimental procedures for copolymerization of different monomers from PVDF-co-CTFE together with the grafting outcomes.

Procedure	Monomer	$[M]_0$ (M)	[M]/[Cl]	Time (min)	Conv %	wt% graft	Theoretical Mn (g/mol)	dn/dc	Mn (MALS) (g/mol)	Đ
3a	PEGMEA480,<br>tBA	1.56	20<br>20	100	52.3<br>50.6	71.8<br>10.7	694,300	0.026	405,000	1.28
3b	PEGMEA480,<br>tBA	1.570	2.5<br>17.5	30	13.0<br>11.7	9.5<br>10.0	149,500	0.0039	1,330,000	1.81
1	PEGMEA480,<br>tBA	1.56	5.9<br>16.1	1.56	180	75<br>84	466,000	N.A.	N.A.	N.A.
2	PEGMEA480,<br>CEA	1.29	9.8<br>15	1.29	50	36<br>42	337,000	N.A.	N.A.	N.A.
3	PEGMEA480,<br>CEA	1.29	4.9<br>7.5	1.29	50	40<br>45	146,000	N.A.	N.A.	N.A.

In each case 200 ppm Cu and 20 ppm eosin Y relative to the total concentration of monomers were used; conversion % was determined using H-NMR spectroscopy using DMSO-d6 solvent.

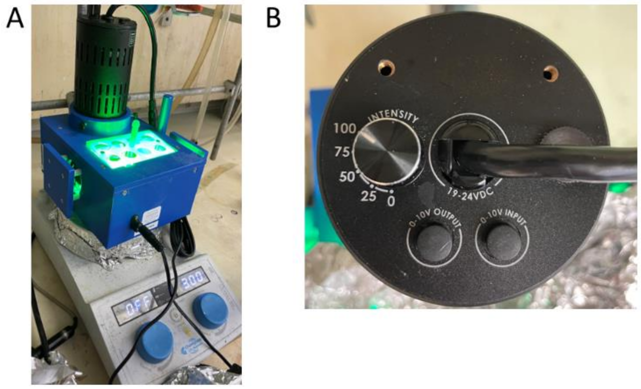

Figure S1. (A) Photochemical reactor set-up with two middle front positions equally exposed to the irradiation and stirring for maintaining homogeneity of viscous solutions, as well as (B) light intensity adjustment.

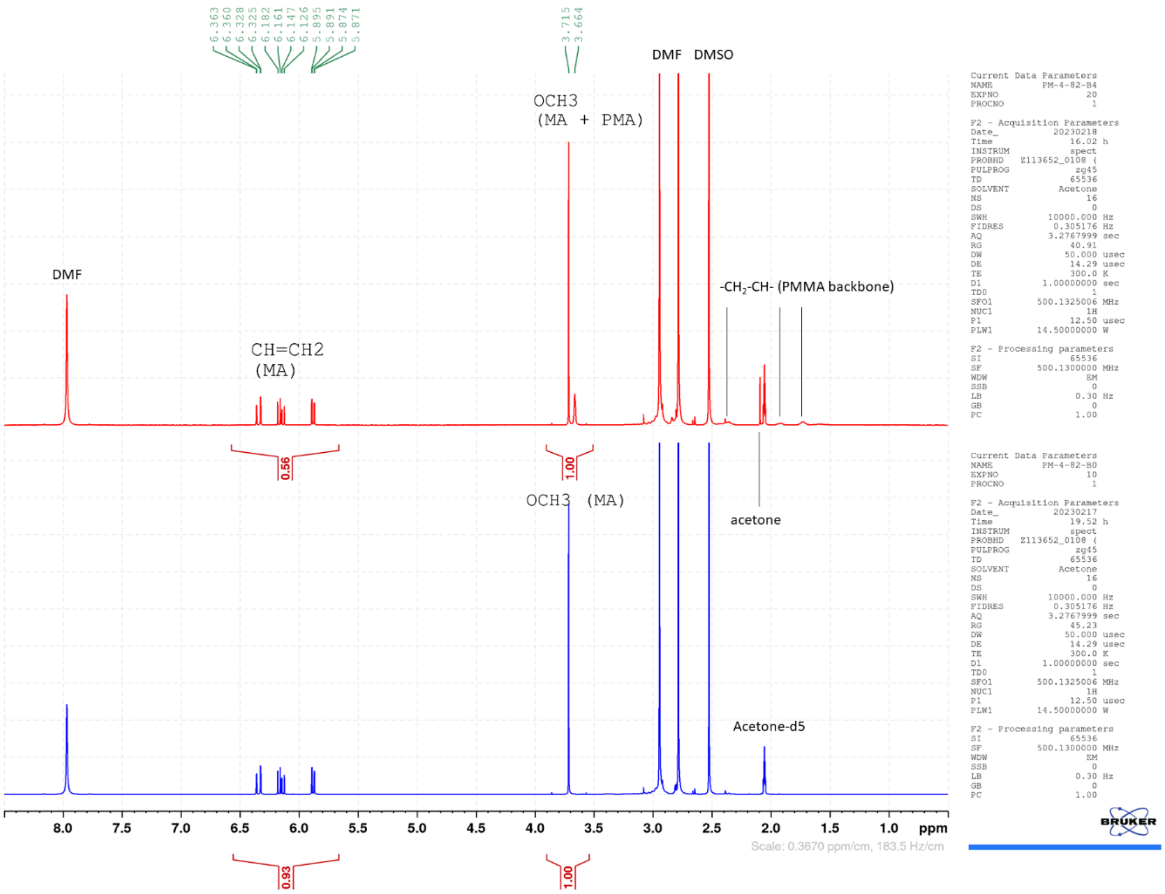

Figure S2. ^{1}H NMR of reaction aliquots from polymerization of MA from PVDF-CTFE (**Table 1**, entry no. 2) at the beginning (bottom) and after 8 h. Measured in acetone-d6. Conversion is calculated from integrals of vinyl proton signals at 5.80-6.44 ppm normalized to methyl ester signals at 3.50-3.91 ppm (OCH_3). Conversion = (0.56/0.93-1)·100% = 40%.

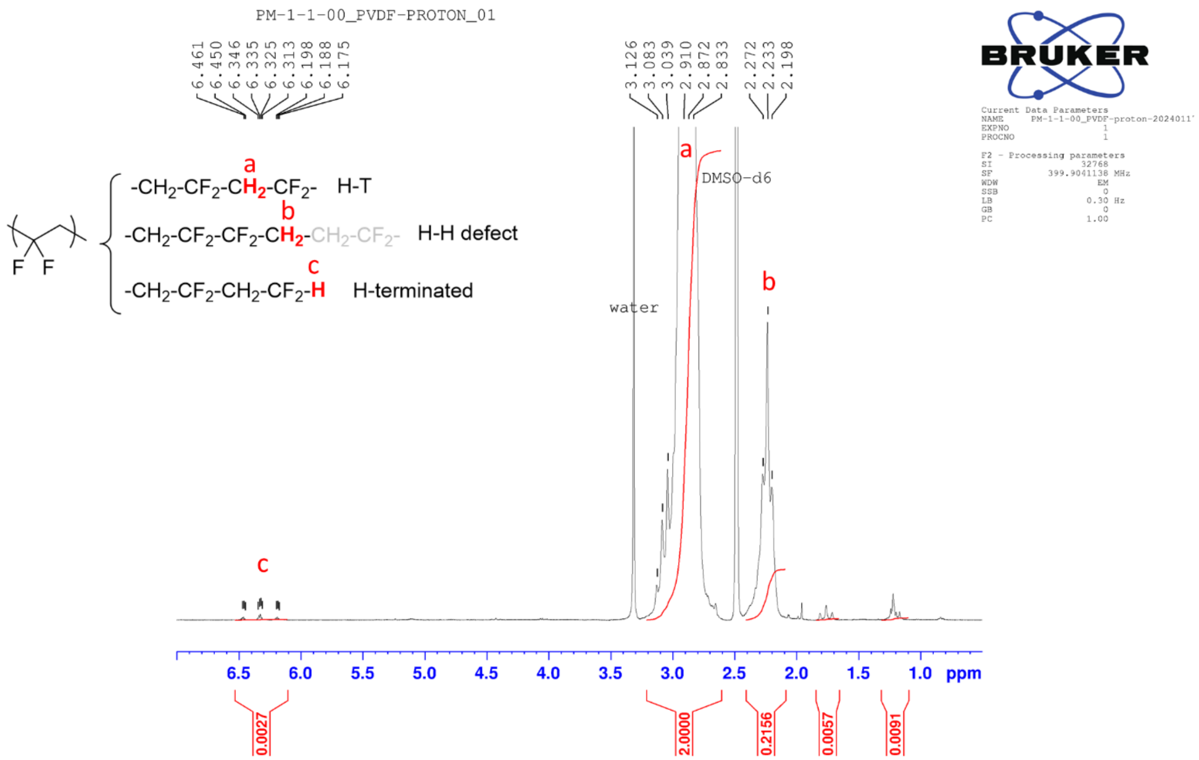

Figure S3. ^{1}H NMR of pristine PVDF. Measured in DMSO-d6 over 256 scans. Signals were assigned according to the literature.[2]

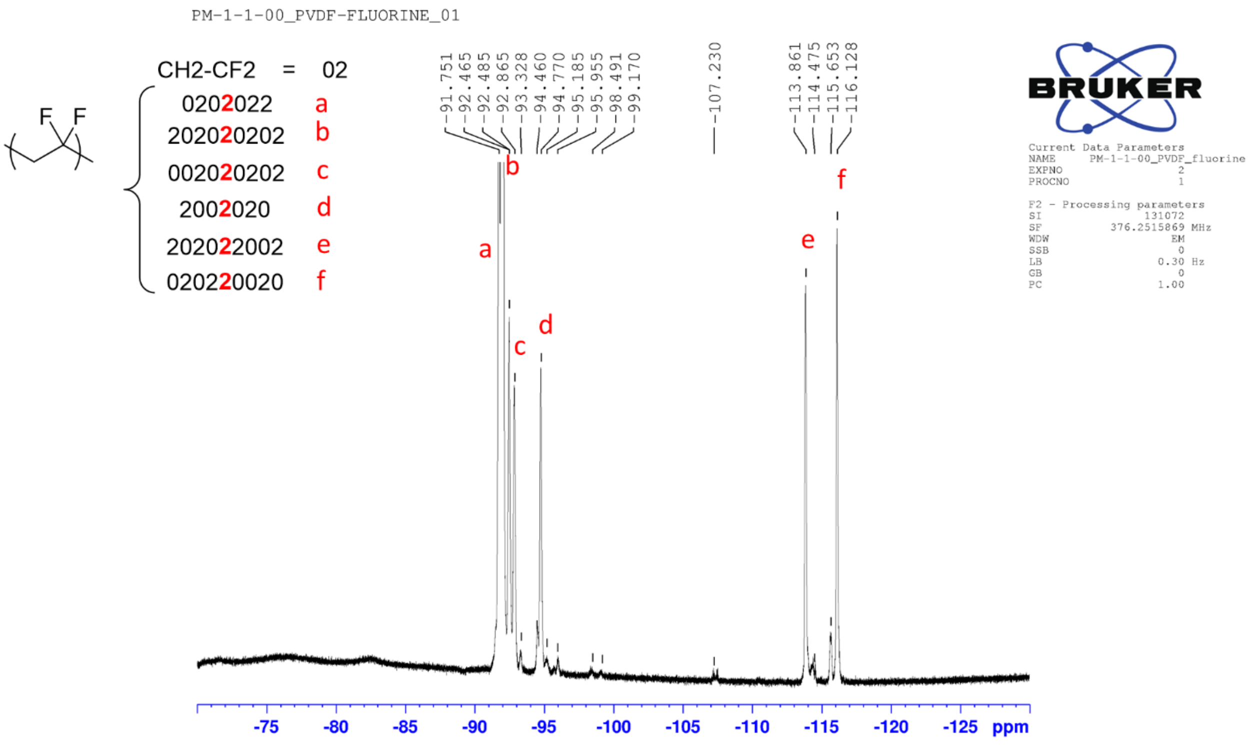

Figure S4. ^{19}F NMR of pristine PVDF. Measured in DMSO-d6 over 128 scans. Signals were assigned according to the literature.[2]

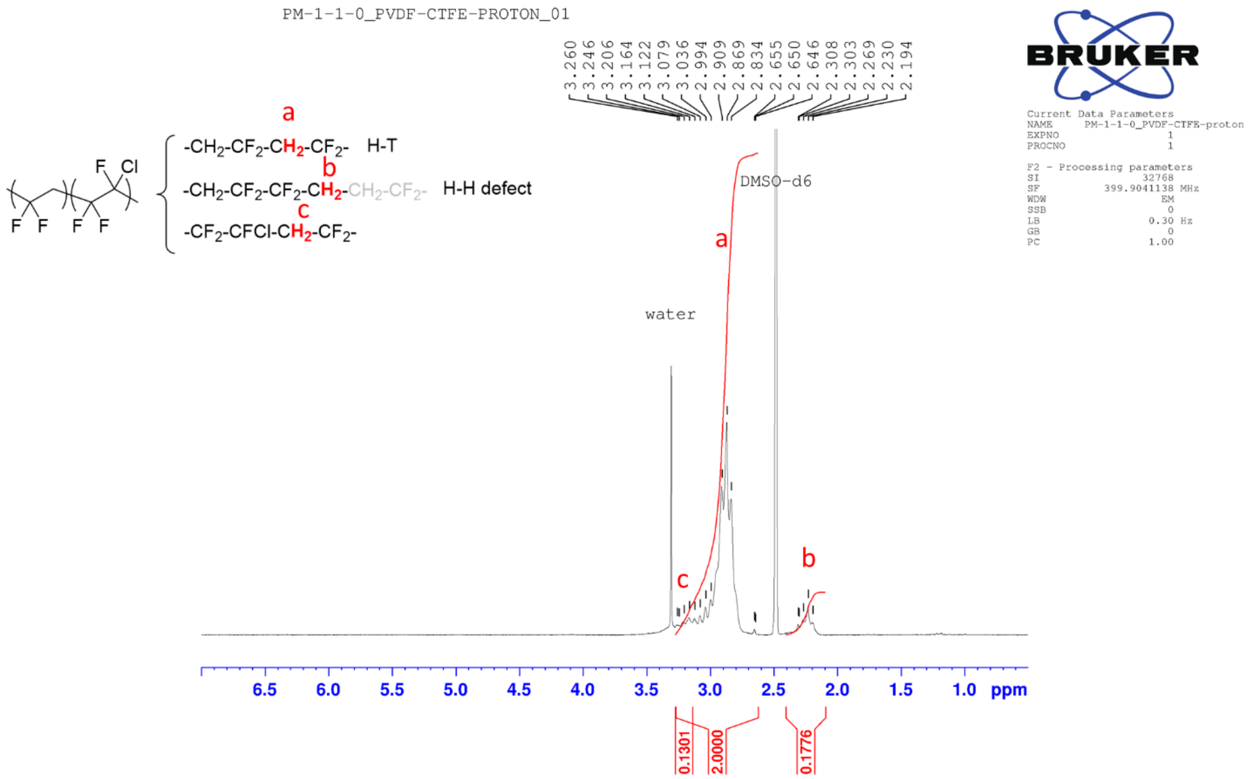

Figure S5. ^{1}H NMR of pristine PVDF-CTFE. Measured in DMSO-d6 over 256 scans. Signals were assigned according to the literature.[3]

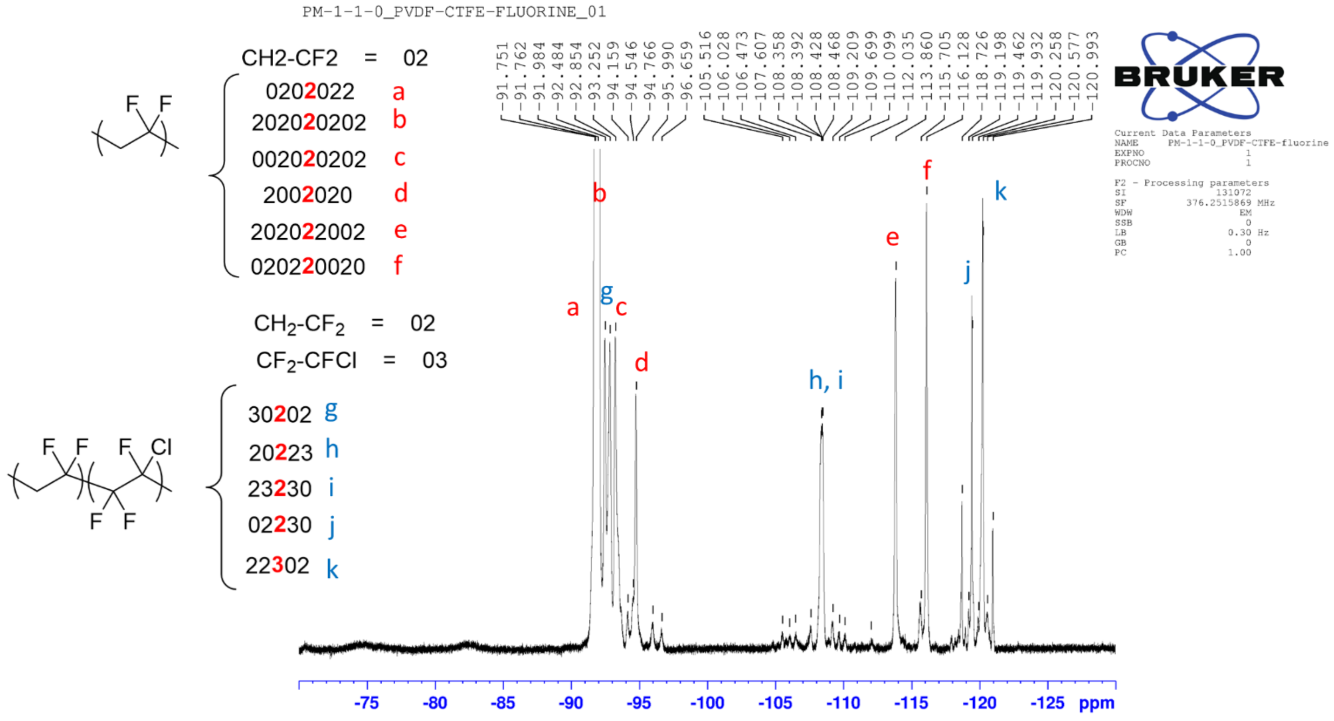

Figure S6. ^{19}F NMR of pristine PVDF. Measured in DMSO-d6 over 128 scans. Signals were assigned according to the literature.[3]

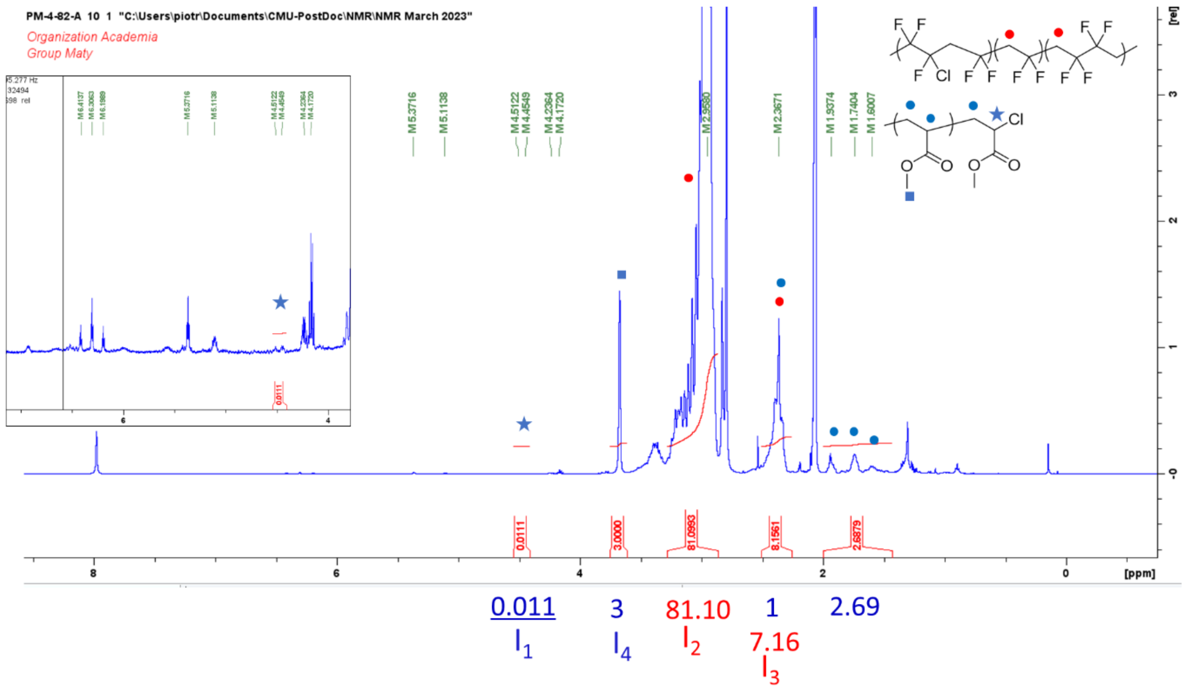

Figure S7. ^{1}H NMR of purified and dried PVDF-CTFE-g-PMA (**Table 1**, entry no. 1). Measured in acetone-d6 over 256 scans. Signal at 2.36 ppm is overlapped by CH_2CF_2 head-to-head defect and PMA backbone signals. The inset is a zoomed area with a visible chain-end signal at 4.45, 4.51 ppm (CH-Cl). DP_{NMR} of PMA was calculated as $I_4/(3I_1)=1/0.011=91$. Grafting density was calculated as $N \cdot I_1/(0.5 \cdot I_2+0.5 \cdot I_3) = 1559 \cdot 0.011/(0.5 \cdot 81.10+0.5 \cdot 7.16) = 0.4$ chains per PVDF-CTFE chain (number of monomer units in PVDF-CTFE chain: $N = M_{n,PVDF-CTFE}/MW_{unit}=108{,}000/69.27=1559$ monomer units; average molecular weight of a monomer unit in PVDF-CTFE chain: $MW_{unit} = (1-\varphi_{CTFE}) \cdot MW_{VDF} + \varphi_{CTFE} \cdot MW_{CTFE} = 0.9 \cdot 64.03+0.1 \cdot 116.47=69.27$ g/mol).

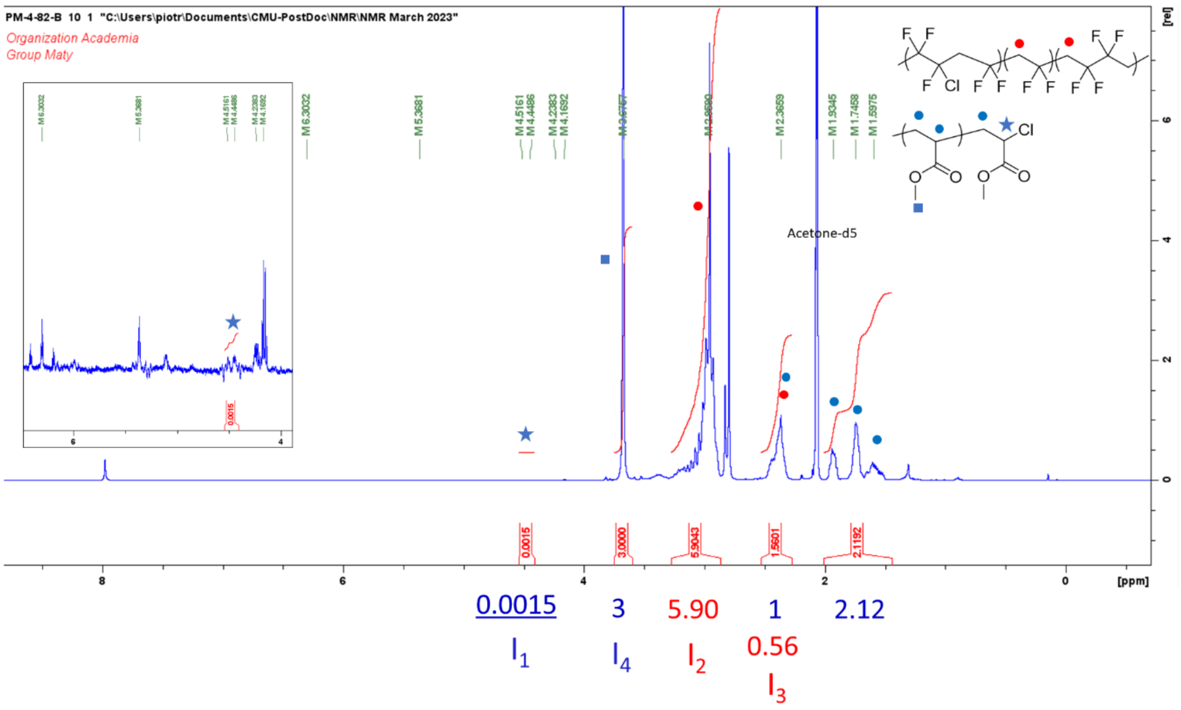

Figure S8. ^{1}H NMR of purified and dried PVDF-CTFE-g-PMA (**Table 1**, entry no. 2). Measured in acetone-d6 over 256 scans. Signal at 2.37 ppm is overlapped by CH_2CF_2 head-to-head defect and PMA backbone signals. The inset is a zoomed area with a visible chain-end signal at 4.45, 4.52 ppm (CH-Cl). DP_{NMR} of PMA was calculated as $I_4/(3I_1)=1/0.0015=667$. Grafting density was calculated as $N \cdot I_1/(0.5 \cdot I_2+0.5 \cdot I_3) = 1559 \cdot 0.0015/(0.5 \cdot 5.90+0.5 \cdot 0.56)=0.7$ chains per PVDF-CTFE chain (number of monomer units in PVDF-CTFE chain: $N = M_{n,PVDF\text{-}CTFE}/MW_{unit}=108{,}000/69.27=1559$ monomer units; average molecular weight of a monomer unit in PVDF-CTFE chain: $MW_{unit} = (1-\varphi_{CTFE}) \cdot MW_{VDF} + \varphi_{CTFE} \cdot MW_{CTFE} = 0.9 \cdot 64.03+0.1 \cdot 116.47=69.27$ g/mol).

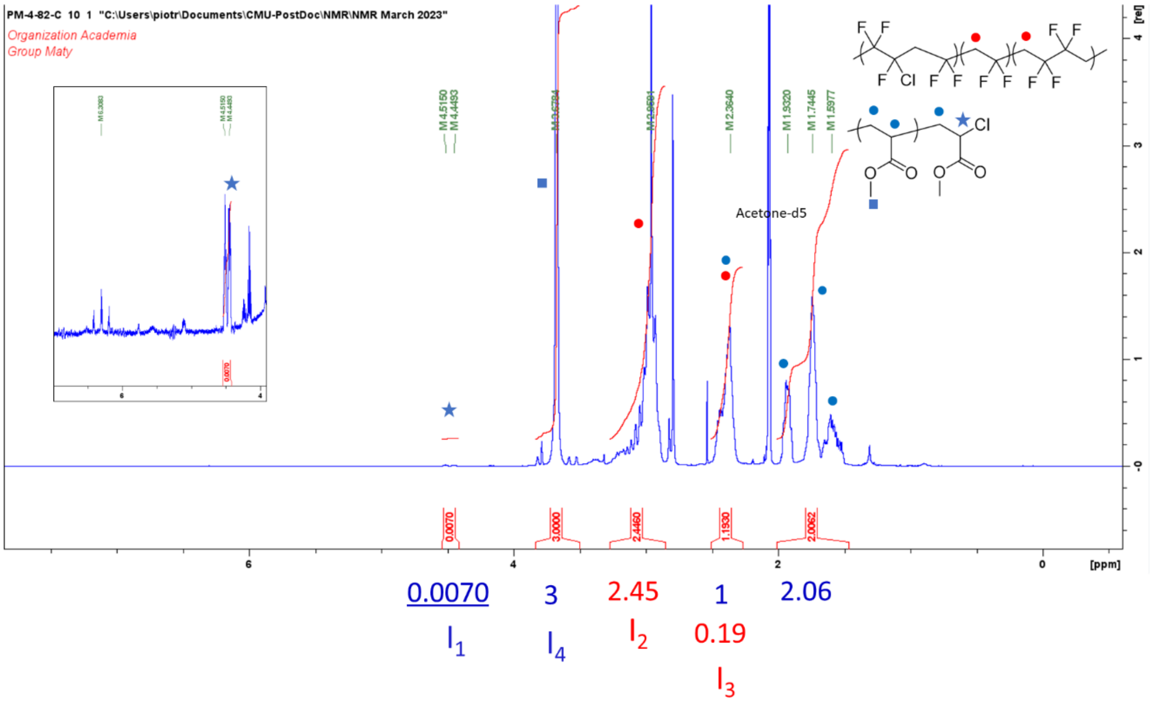

Figure S9. ^{1}H NMR of purified and dried PVDF-CTFE-g-PMA (**Table 1**, entry no. 3). Measured in acetone-d6 over 256 scans. Signal at 2.36 ppm is overlapped by CH_2CF_2 head-to-head defect and PMA backbone signals. The inset is a zoomed area with a visible chain-end signal at 4.45, 4.52 ppm (CH-Cl). DP_{NMR} of PMA was calculated as $I_4/(3I_1)=1/0.0070=143$. Grafting density was calculated as $N \cdot I_1/(0.5 \cdot I_2+0.5 \cdot I_3) \cdot N = 1559 \cdot 0.0070/(0.5 \cdot 2.45+0.5 \cdot 0.19)=8.3$ chains per PVDF-CTFE chain (number of monomer units in PVDF-CTFE chain: $N = M_{n,PVDF-CTFE}/MW_{unit}=108{,}000/69.27=1559$ monomer units; average molecular weight of a monomer unit in PVDF-CTFE chain: $MW_{unit} = (1-\varphi_{CTFE}) \cdot MW_{VDF} + \varphi_{CTFE} \cdot MW_{CTFE} = 0.9 \cdot 64.03+0.1 \cdot 116.47=69.27$ g/mol).

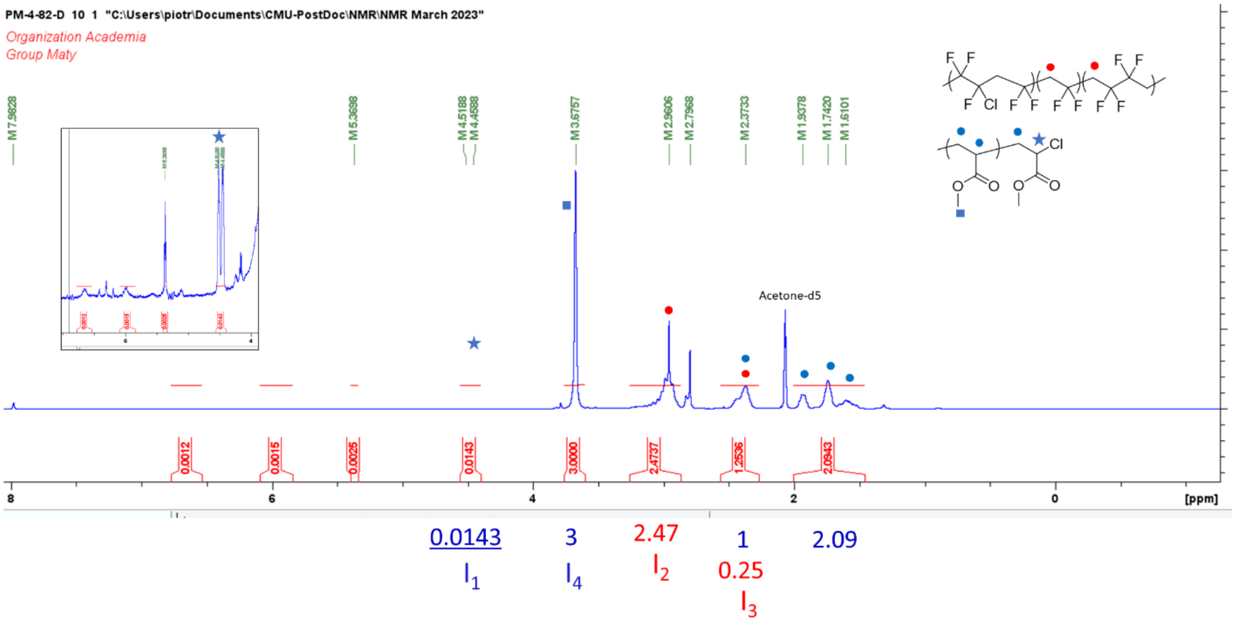

Figure S10. ^{1}H NMR of purified and dried PVDF-CTFE-g-PMA (**Table 1**, entry no. 4). Measured in acetone-d6 over 256 scans. Signal at 2.36 ppm is overlapped by CH_2CF_2 head-to-head defect and PMA backbone signals. The inset is a zoomed area with a visible chain-end signal at 4.46, 4.52 ppm (CH-Cl). DP_{NMR} of PMA was calculated as $I_4/(3I_1)=1/0.0143=70$. Grafting density was calculated as $N \cdot I_1/(0.5 \cdot I_2+0.5 \cdot I_3) \cdot N = 1559 \cdot 0.0143/(0.5 \cdot 2.47+0.5 \cdot 0.25)=16.4$ chains per PVDF-CTFE chain (number of monomer units in PVDF-CTFE chain: $N = M_{n,PVDF\text{-}CTFE}/MW_{unit}=108{,}000/69.27=1559$ monomer units; average molecular weight of a monomer unit in PVDF-CTFE chain: $MW_{unit} = (1-\varphi_{CTFE}) \cdot MW_{VDF} + \varphi_{CTFE} \cdot MW_{CTFE} = 0.9 \cdot 64.03+0.1 \cdot 116.47=69.27$ g/mol).

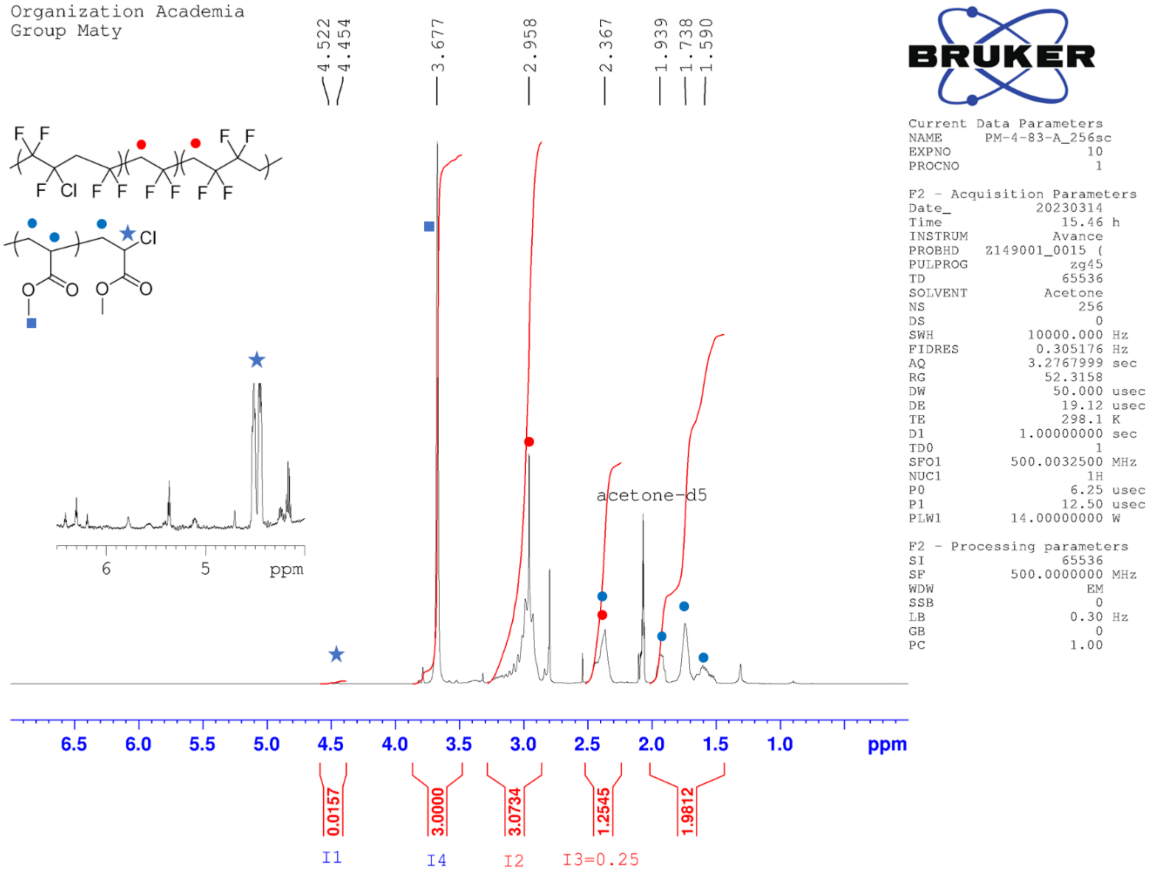

Figure S11. ^{1}H NMR of purified and dried PVDF-CTFE-g-PMA (**Table 1**, entry no. 5). Measured in acetone-d6 over 256 scans. Signal at 2.36 ppm is overlapped by CH_2CF_2 head-to-head defect and PMA backbone signals. The inset is a zoomed area with a visible chain-end signal at 4.44, 4.52 ppm (CH-Cl). DP_{NMR} of PMA was calculated as $I_4/(3I_1)=1/0.0157=64$. Grafting density was calculated as $N \cdot I_1/(0.5 \cdot I_2+0.5 \cdot I_3) = 1559 \cdot 0.0157/(0.5 \cdot 3.07+0.5 \cdot 0.25) = 15.1$ chains per PVDF-CTFE chain (number of monomer units in PVDF-CTFE chain: $N = M_{n,PVDF-CTFE}/MW_{unit}=108{,}000/69.27=1559$ monomer units; average molecular weight of a monomer unit in PVDF-CTFE chain: $MW_{unit} = (1-\varphi_{CTFE}) \cdot MW_{VDF} + \varphi_{CTFE} \cdot MW_{CTFE} = 0.9 \cdot 64.03+0.1 \cdot 116.47=69.27$ g/mol).

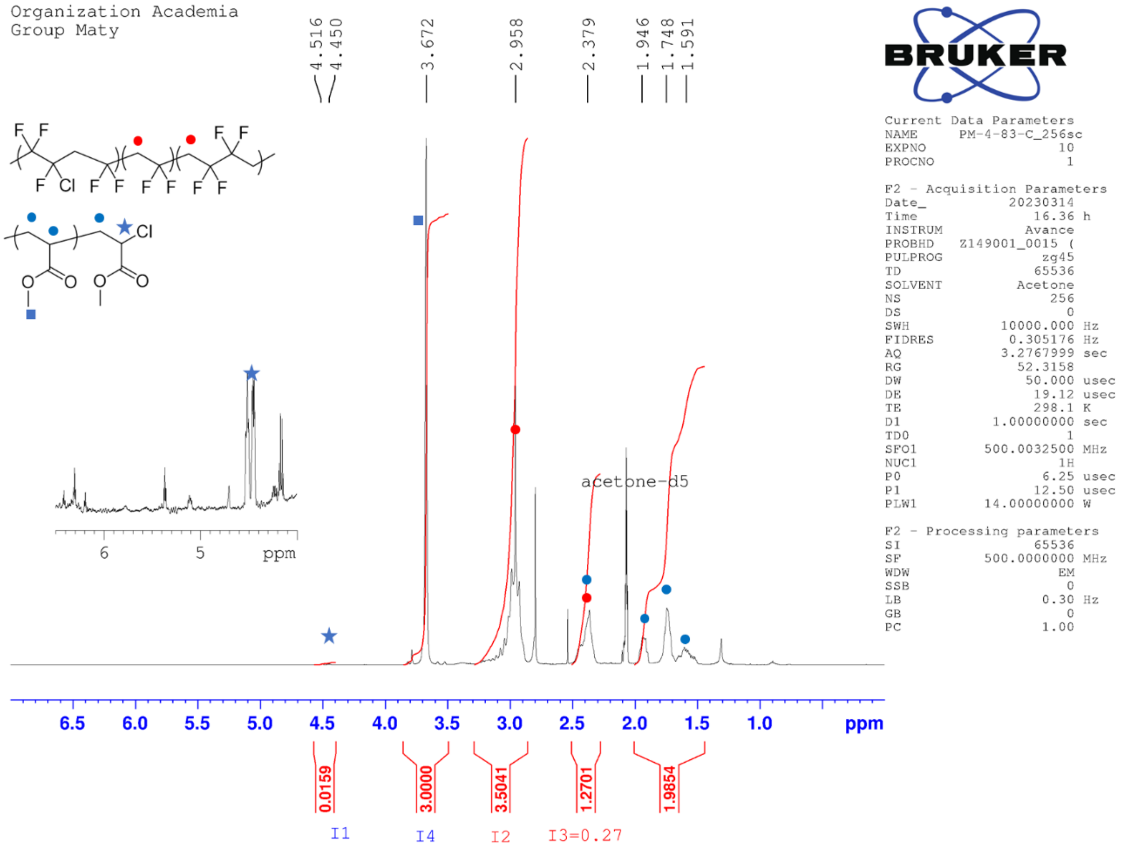

Figure S12. ^{1}H NMR of purified and dried PVDF-CTFE-g-PMA (**Table 1**, entry no. 7). Measured in acetone-d6 over 256 scans. Signal at 2.36 ppm is overlapped by CH_2CF_2 head-to-head defect and PMA backbone signals. The inset is a zoomed area with a visible chain-end signal at 4.45, 4.52 ppm (CH-Cl). DP_{NMR} of PMA was calculated as $I_4/(3I_1)=1/0.0159=63$. Grafting density was calculated as $N \cdot I_1/(0.5 \cdot I_2+0.5 \cdot I_3) = 1559 \cdot 0.0159/(0.5 \cdot 3.50+0.5 \cdot 0.27) = 0.4$ chains per PVDF-CTFE chain Grafting density is calculated as $I_1/(0.5 \cdot I_2+0.5 \cdot I_3) \cdot N = 0.0159/(0.5 \cdot 3.50+0.5 \cdot 0.27) \cdot 1559=13.2$ chains per PVDF-CTFE chain (number of monomer units in PVDF-CTFE chain: $N = M_{n,PVDF\text{-}CTFE}/MW_{unit}=108{,}000/69.27=1559$ monomer units; average molecular weight of a monomer unit in PVDF-CTFE chain: $MW_{unit} = (1-\varphi_{CTFE}) \cdot MW_{VDF} + \varphi_{CTFE} \cdot MW_{CTFE} = 0.9 \cdot 64.03+0.1 \cdot 116.47=69.27$ g/mol).

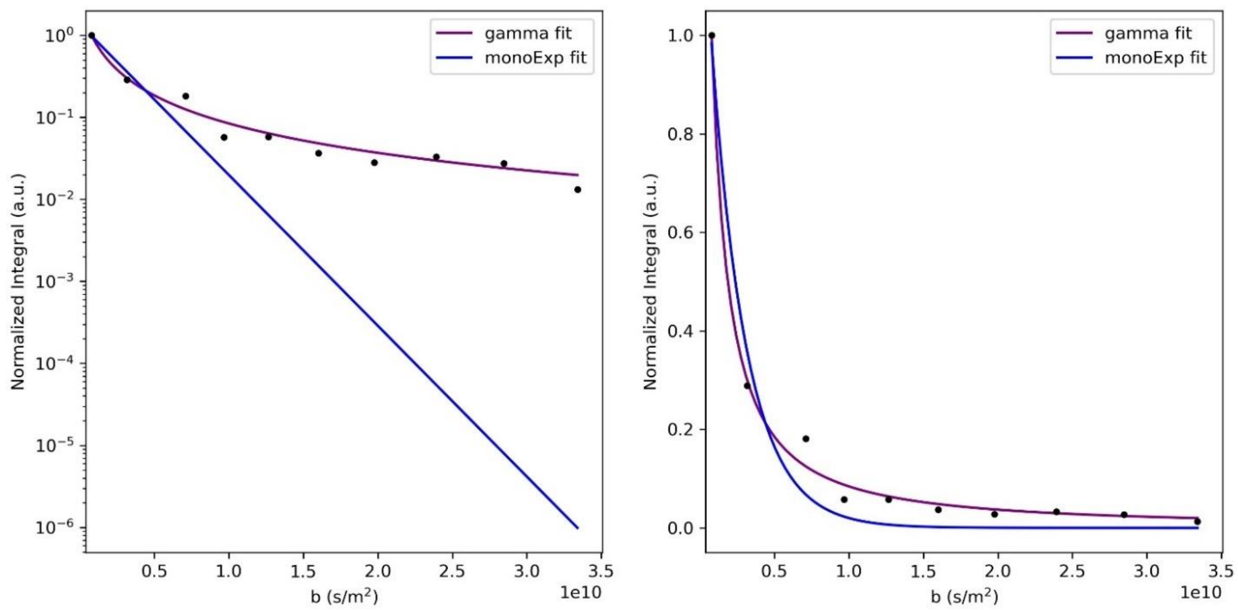

Figure S13. Comparison of the gamma model with the monoexponential model for PVDF sample.

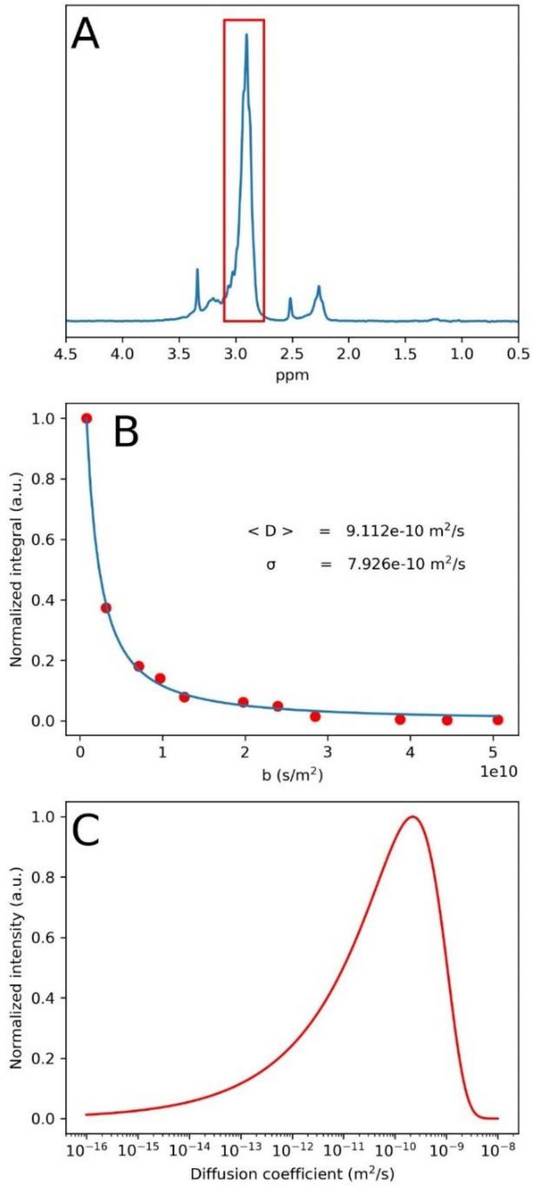

Figure S14. DOSY NMR analysis of PVDF-CTFE; the backbone (CH_2-CF_2 signal at 2.98 ppm, red). (A) 1D ^{1}H NMR spectrum of PVDF-CTFE. (B) Quality fit of the gamma-model for the CH_2-CF_2. (C) Diffusion coefficient distribution of the peak. DOSY NMR was carried out in DMSO-d6.

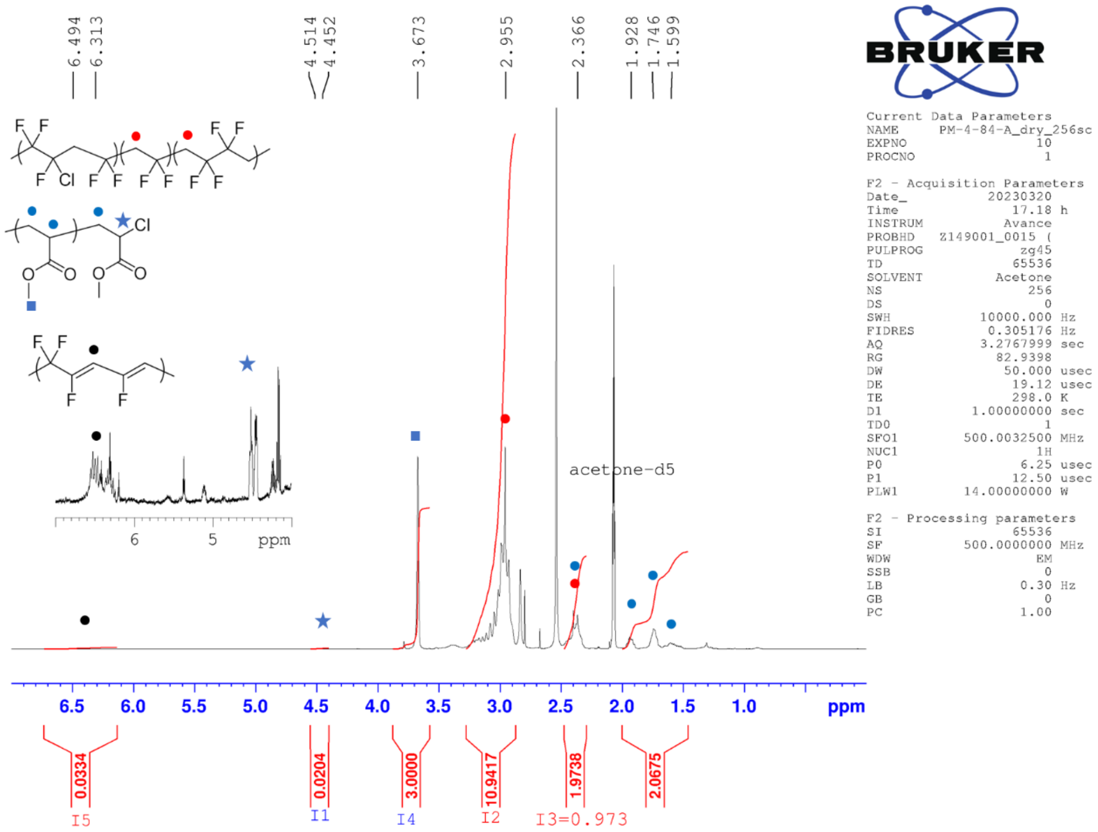

Figure S15. ^{1}H NMR of purified and dried PVDF-CTFE-g-PMA (**Table 2**, entry no. 2). Measured in acetone-d6 over 256 scans. Signal at 2.37 ppm is overlapped by CH_2CF_2 head-to-head defect and PMA backbone signals. The inset is a zoomed area with a visible chain-end signal at 4.45, 4.51 ppm (CH-Cl). DP_{NMR} of PMA was calculated as $I_4/(3I_1)=1/0.0204=49$. Grafting density was calculated as $N \cdot I_1/(0.5 \cdot I_2+0.5 \cdot I_3) = 1559 \cdot 0.0204/(0.5 \cdot 10.94+0.5 \cdot 0.974) = 5.3$ chains per PVDF-CTFE chain (number of monomer units in PVDF-CTFE chain: $N = M_{n,PVDF\text{-}CTFE}/MW_{unit}=108{,}000/69.27=1559$ monomer units; average molecular weight of a monomer unit in PVDF-CTFE chain: $MW_{unit} = (1-\varphi_{CTFE}) \cdot MW_{VDF} + \varphi_{CTFE} \cdot MW_{CTFE} = 0.9 \cdot 64.03+0.1 \cdot 116.47=69.27$ g/mol).

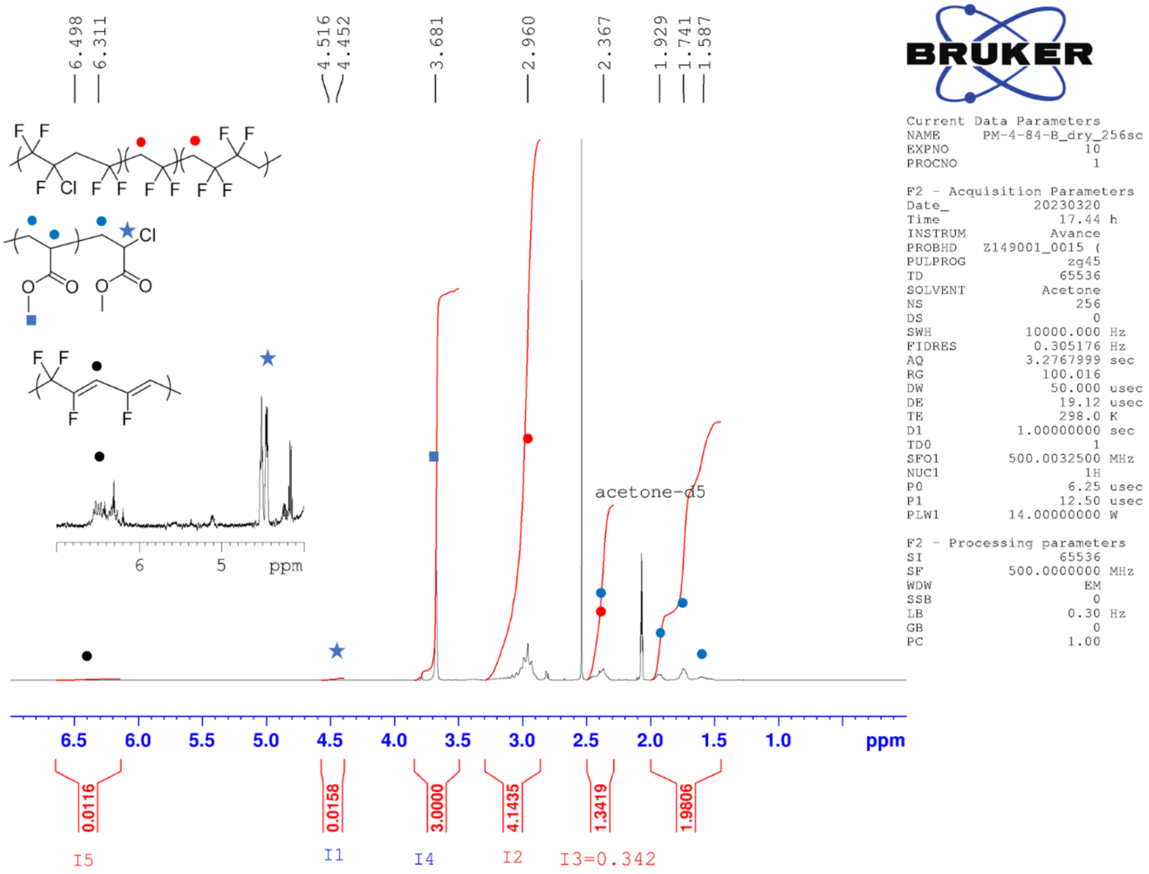

Figure S16. ^{1}H NMR of purified and dried PVDF-CTFE-g-PMA (**Table 2**, entry no. 4). Measured in acetone-d6 over 256 scans. Signal at 2.37 ppm is overlapped by CH_2CF_2 head-to-head defect and PMA backbone signals. The inset is a zoomed area with a visible chain-end signal at 4.45, 4.52 ppm (CH-Cl). DP_{NMR} of PMA was calculated as $I_4/(3I_1)=1/0.0158=63$. Grafting density was calculated as $N \cdot I_1/(0.5 \cdot I_2+0.5 \cdot I_3)$ = $1559 \cdot 0.0158/(0.5 \cdot 4.14+0.5 \cdot 0.342)$ = 11.0 chains per PVDF-CTFE chain (number of monomer units in PVDF-CTFE chain: $N = M_{n,PVDF\text{-}CTFE}/MW_{unit}=108{,}000/69.27=1559$ monomer units; average molecular weight of a monomer unit in PVDF-CTFE chain: $MW_{unit} = (1-\varphi_{CTFE}) \cdot MW_{VDF} + \varphi_{CTFE} \cdot MW_{CTFE} = 0.9 \cdot 64.03+0.1 \cdot 116.47=69.27$ g/mol).

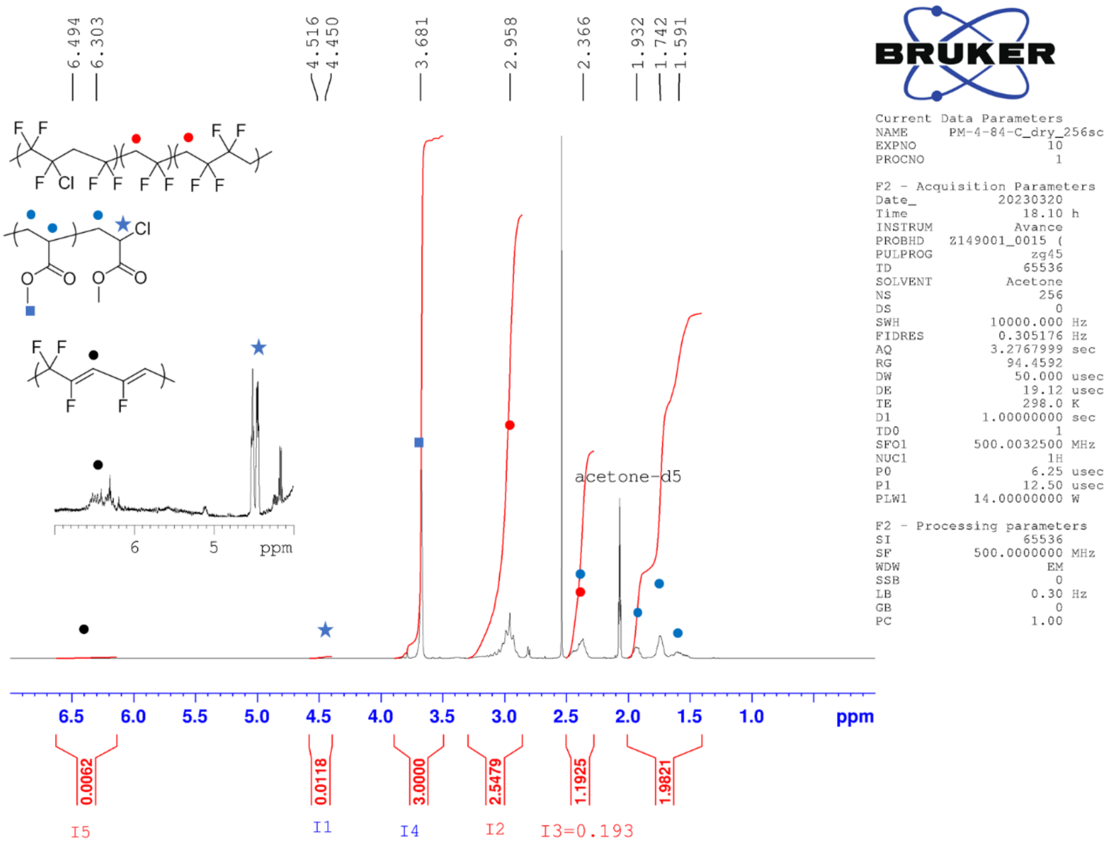

Figure S17. ^{1}H NMR of purified and dried PVDF-CTFE-g-PMA (**Table 2**, entry no. 6). Measured in acetone-d6 over 256 scans. Signal at 2.37 ppm is overlapped by CH_2CF_2 head-to-head defect and PMA backbone signals. The inset is a zoomed area with a visible chain-end signal at 4.45, 4.52 ppm (CH-Cl). DP_{NMR} of PMA was calculated as $I_4/(3I_1)=1/0.0118=85$. Grafting density was calculated as $N \cdot I_1/(0.5 \cdot I_2+0.5 \cdot I_3)$ = $1559 \cdot 0.0118/(0.5 \cdot 2.55+0.5 \cdot 0.193)$ = 15.6 chains per PVDF-CTFE chain (number of monomer units in PVDF-CTFE chain: $N = M_{n,PVDF\text{-}CTFE}/MW_{unit}=108{,}000/69.27=1559$ monomer units; average molecular weight of a monomer unit in PVDF-CTFE chain: $MW_{unit} = (1-\varphi_{CTFE}) \cdot MW_{VDF} + \varphi_{CTFE} \cdot MW_{CTFE} = 0.9 \cdot 64.03+0.1 \cdot 116.47=69.27$ g/mol).

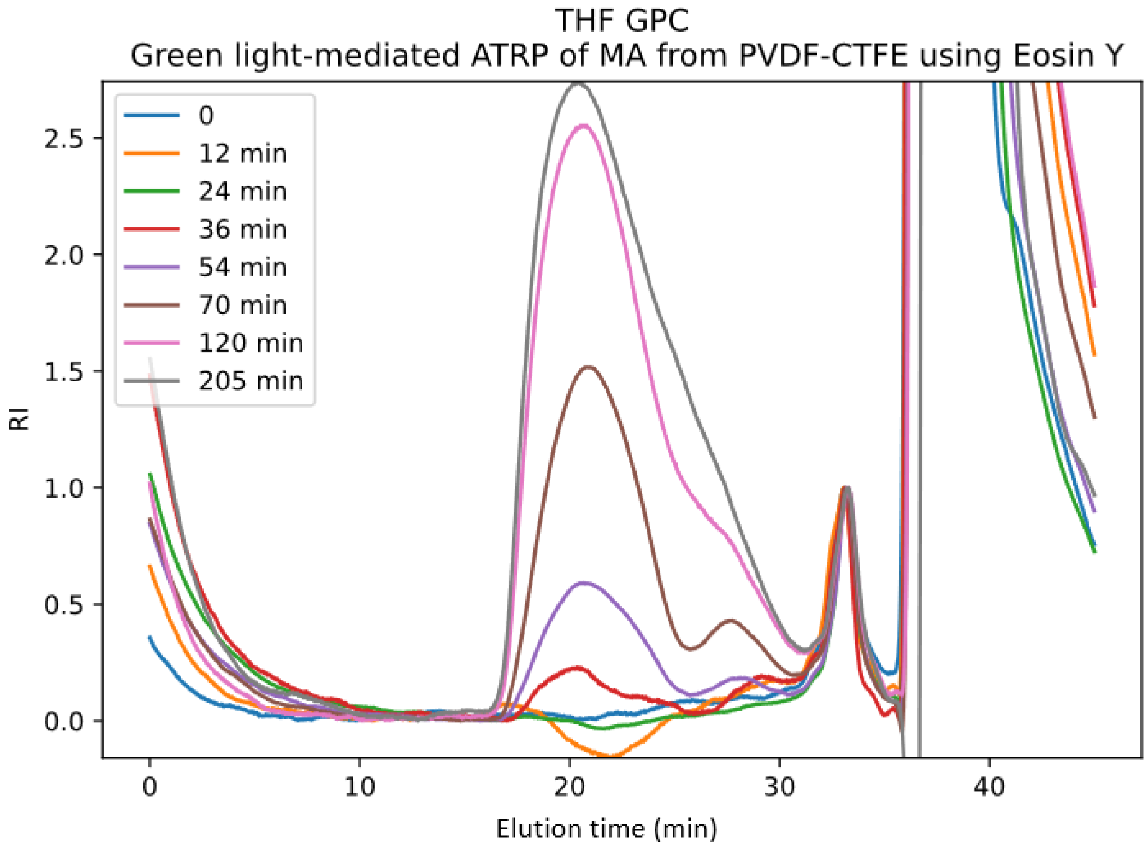

Figure S18. GPC traces (THF) of aliquots from Photo-ATRP of MA from PVDF-CTFE. Signal at 20 min is PVDF-CTFE, while signal at ca. 32 min is the MA monomer.

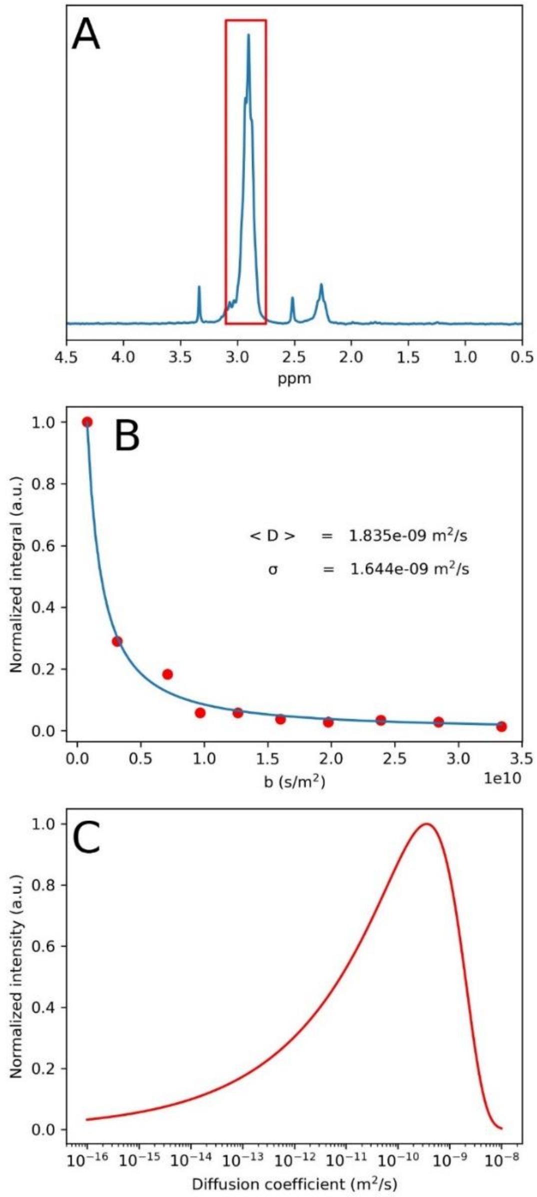

Figure S19. DOSY NMR analysis of PVDF; the backbone (CH_2-CF_2 signal at 2.98 ppm, red). (A) 1D ^{1}H NMR spectrum of PVDF. (B) Quality fit of the gamma-model for the CH_2-CF_2. (C) Diffusion coefficient distribution of the peak. DOSY NMR was carried out in DMSO-d6.

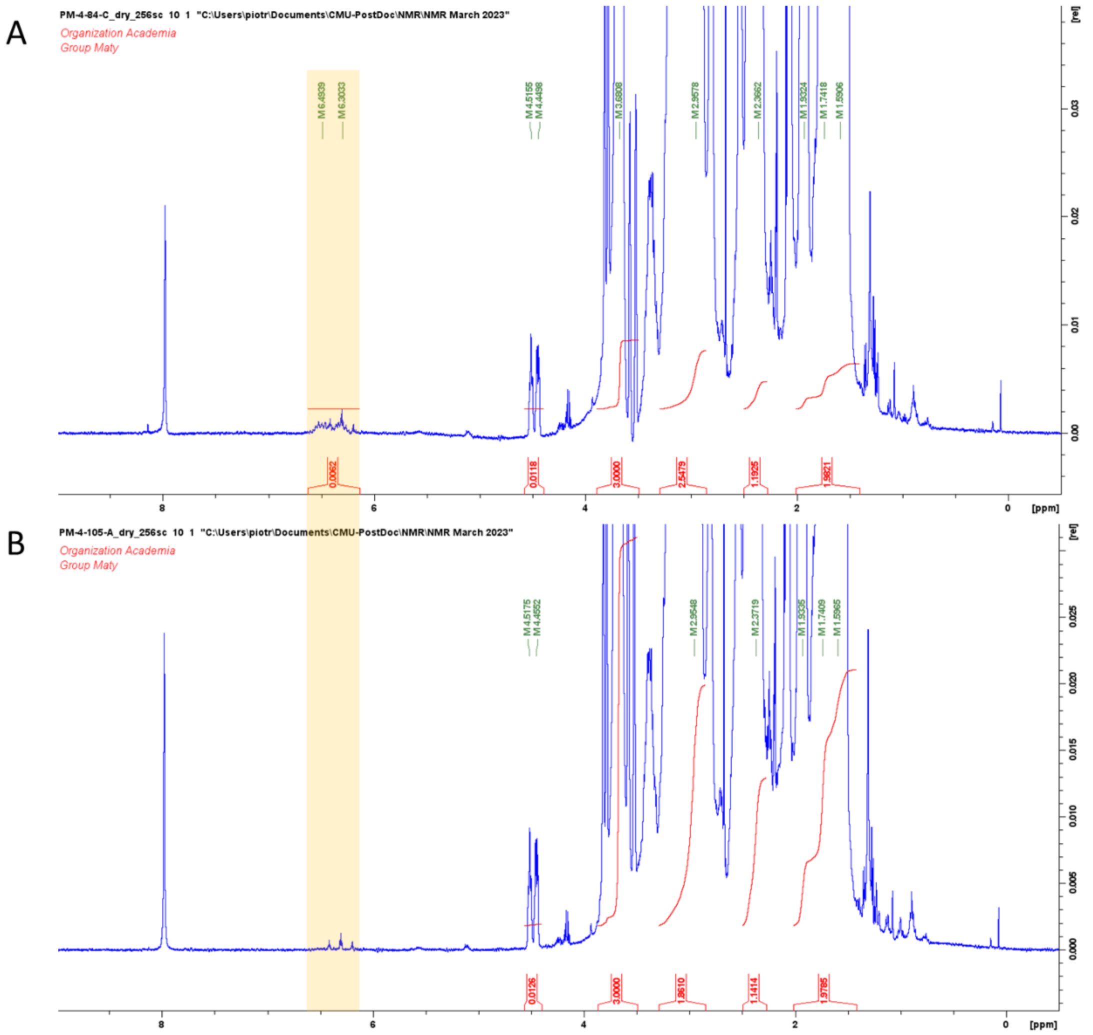

Figure S20. ^{1}H NMR spectra of dry PVDF-CTFE-g-PMA samples obtained using experimental procedures with (**A**) 12 equivalents and (**B**) 3 equivalents of Me6TREN. The yellow shaded region covers vinyl proton area. 12 equivalents of Me_6TREN clearly led to formation of more unsaturations (6.49-6.30 ppm). The triplet signal at 6.31 ppm is the same as in unmodified, as received PVDF-CTFE. Conditions: MA/C-Cl/$CuCl_2$/Me_6TREN/EY·Na_2=20/1/0.004/0.012(or 0.048)/0.0004; DMSO/DMF = 9/1, v/v; [MA] = 2 M, [$CuCl_2$]/[MA] = 200 ppm, V_{tot} = 4 mL, under green light irradiation in PhotoRedOx Box™ equipped with Kessil LED light (525 nm) under 100% intensity (ca. 25 mW/cm^2) over 1 (**A**) and 2 hours (**B**).

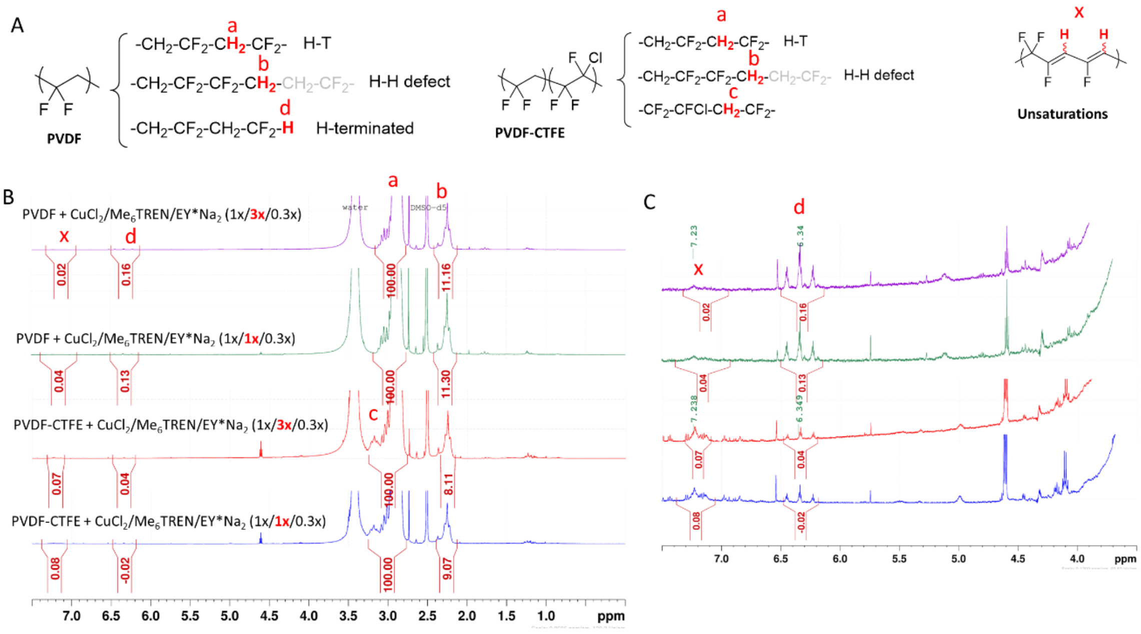

Figure S21. (A) Chemical structures of PVDF, PVDF-CTFE and expected unsaturatated species. (B) ^{1}H NMR spectra of PVDF-CTFE (blue, red) and PVDF (green, violet) purified samples that were exposed for 1 h to solutions mimicking ATRP conditions; with 1 equivalent and 3 equivalents of Me6TREN. (C) Magnified spectra at the vinyl region. The vinyl signals are at 7.30-6.84 ppm. The triplet signal at 6.34 ppm is the same as in unmodified, as received PVDF and PVDF-CTFE. Conditions: C-Cl/$CuCl_2$/Me_6TREN/EY·Na_2=1/0.004/0.004 (or 0.012)/0.0012; DMSO/DMF = 9/1, v/v; V_{tot} = 4 mL. The solutions irradiated with green light produced similar spectra.

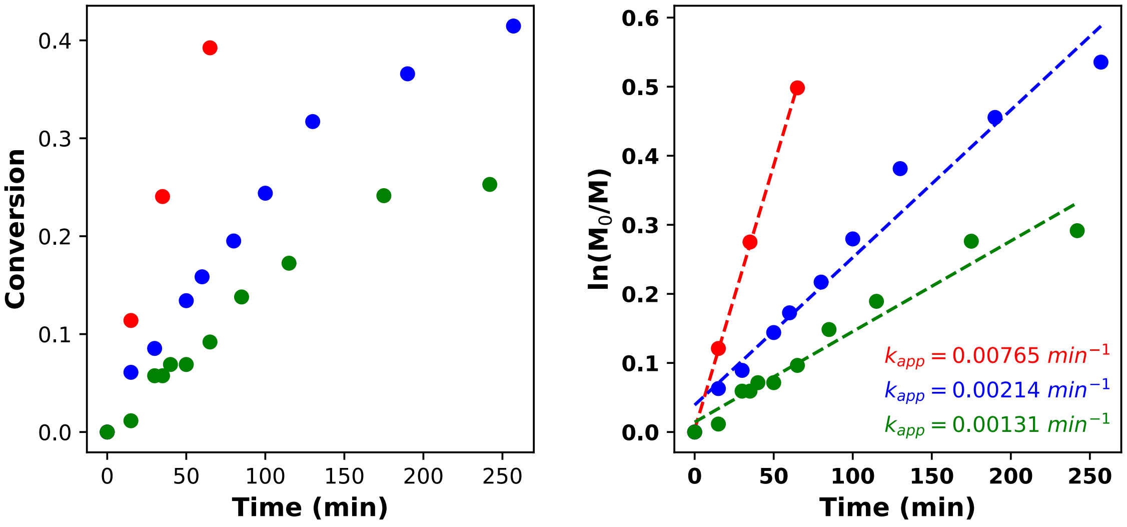

Figure S22. Kinetic plots of grafting PtBA from PVDF-co-CTFE. Conditions as in Table 5, DP=20 (red), DP=40 (blue) and D=P80 (green).

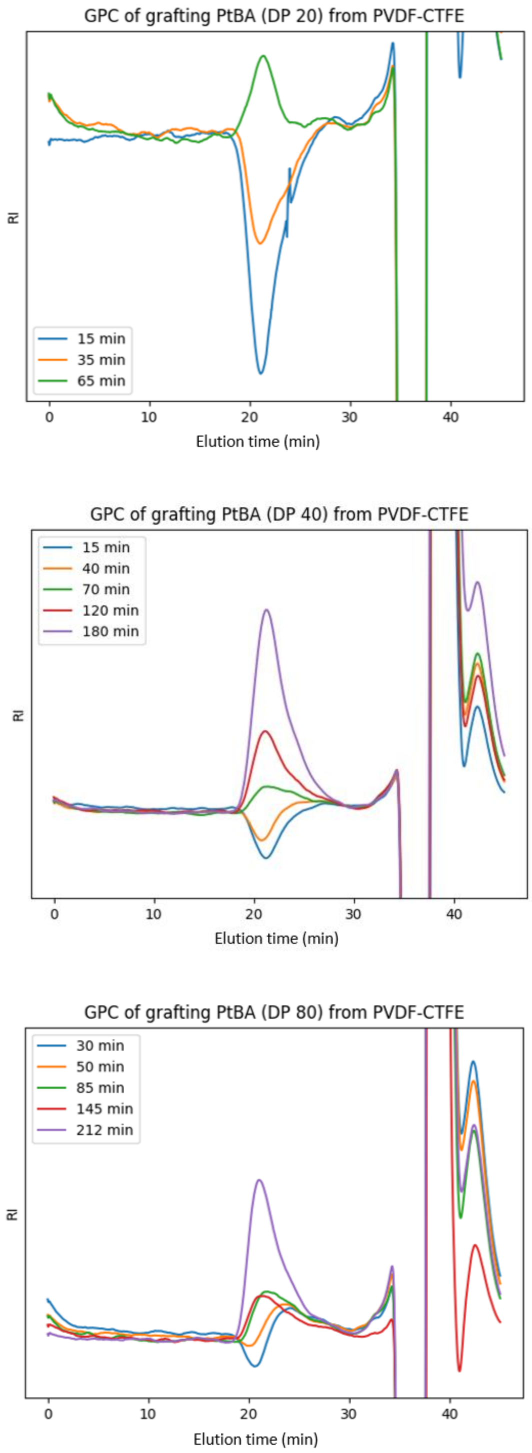

Figure S23. GPC traces of aliquots from polymerization solutions at different time points; [tBA]/[Cl]=20/1 (top), 40/1 (middle) and 80/1 (bottom). THF was used as the eluent.

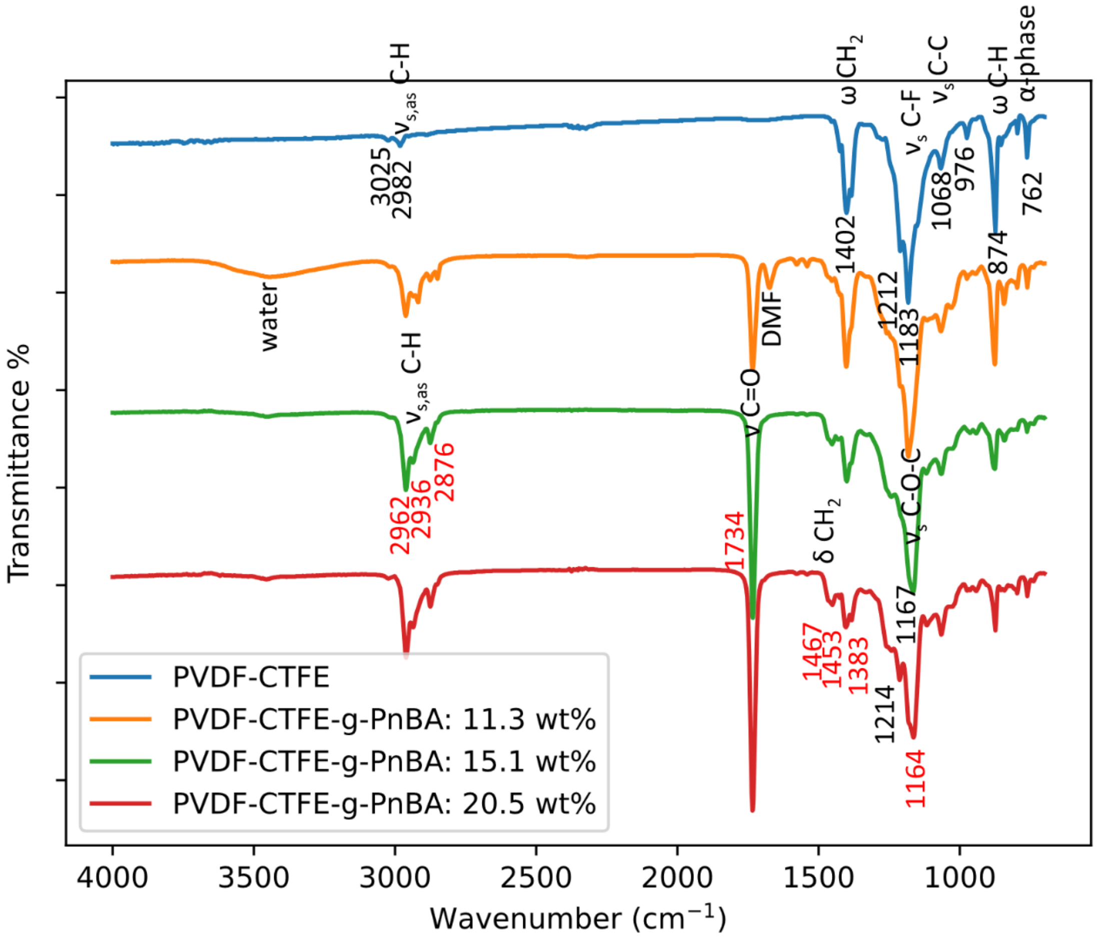

Figure S24. FTIR spectra of pristine PVDF-CTFE and selected grafted samples with PnBA. Stretching ($\nu_{s,as}$ C-H, ν_s C-F, ν_s C-C), wagging (ω CH_2, ω C-H) and crystalline α phase bands of PVDF were assigned according to literature.[4–7] After the grafting, new bands appear characteristic to PnBA. Strong and clear stretching bands, $\nu_{s,as}$ C-H (2962, 2936, 2876 cm^{-1}) and ν C=O (1734 cm^{-1}) are clearly observed. More subtle changes in the fingerprint region were also noted, among others, corresponding to bending (δ CH_2 at 1467-1453 cm^{-1}) and stretching (ν_s C-O-C at 1164 cm^{-1}) bands.[8]

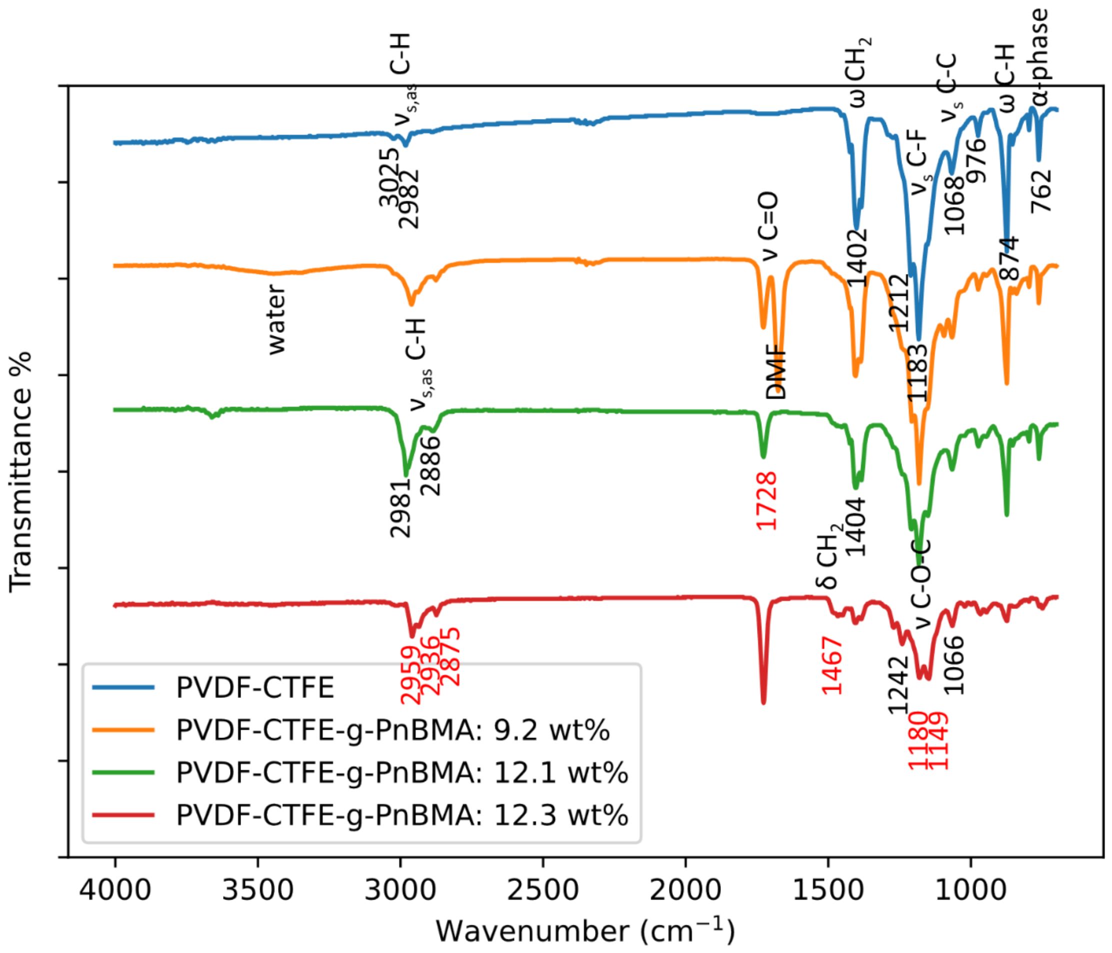

Figure S25. FTIR spectra of pristine PVDF-CTFE and selected grafted samples with PnBMA. Stretching ($\nu_{s,as}$ C-H, ν_s C-F, ν_s C-C), wagging (ω CH_2, ω C-H) and crystalline α phase bands of PVDF were assigned according to literature. [4–7] After the grafting, new bands appear characteristic to PnBA. Strong and clear stretching bands, $\nu_{s,as}$ C-H (2959, 2936, 2875 cm^{-1}) and ν C=O (1728 cm^{-1}) are clearly observed. More subtle changes in the fingerprint region were also noted, among others, corresponding to bending (δ CH_2 at 1467 cm^{-1}) and stretching (ν_s C-O-C at 1180, 1149 cm^{-1}) bands.[9]

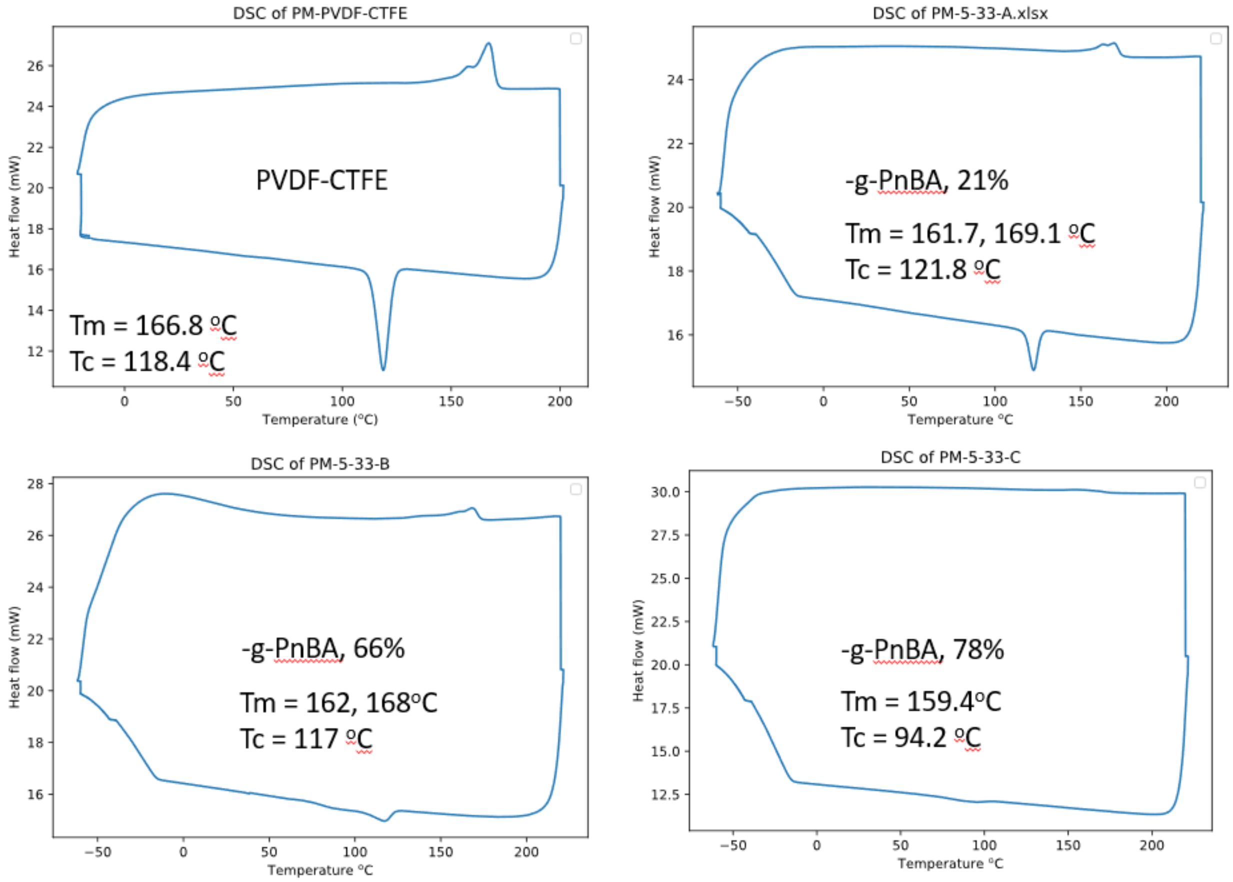

Figure S26. DSC of PVDF-CTFE and PVDF-CTFE grafted with PnBA.

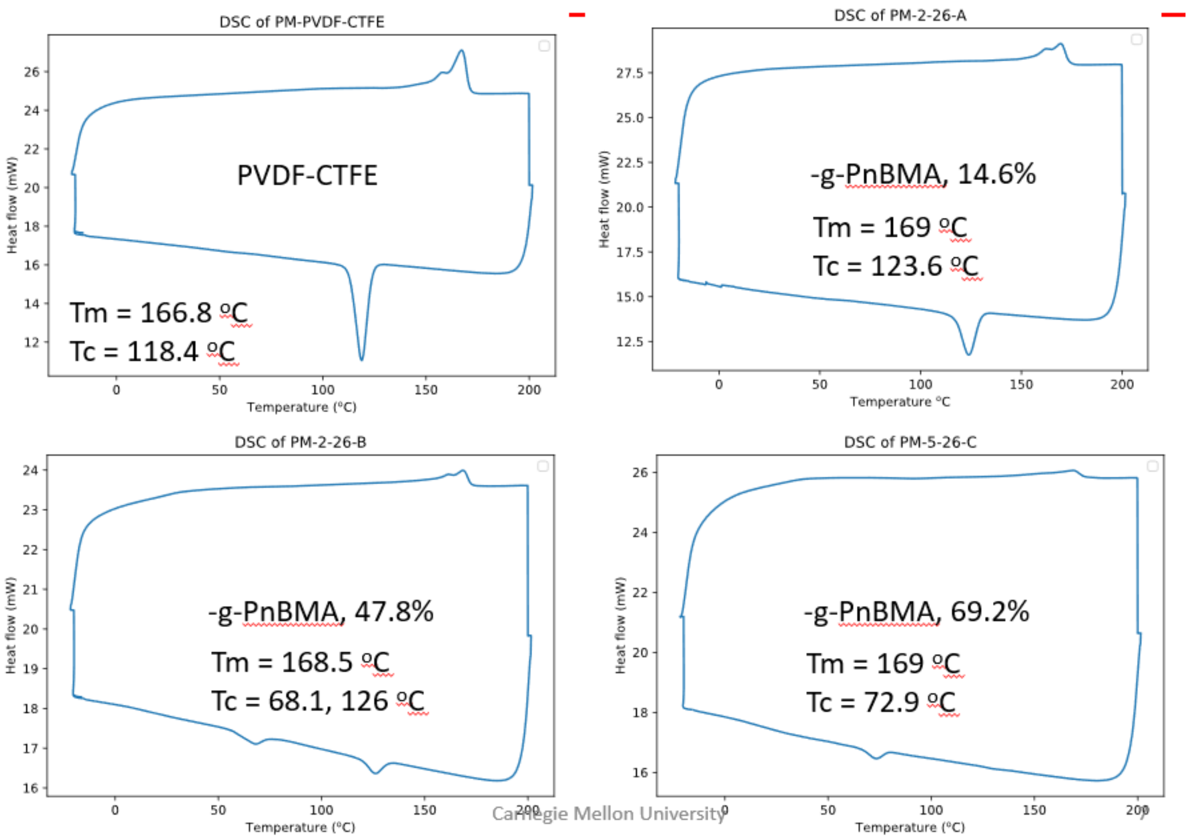

Figure S27. DSC of PVDF-CTFE and PVDF-CTFE grafted with PnBMA.

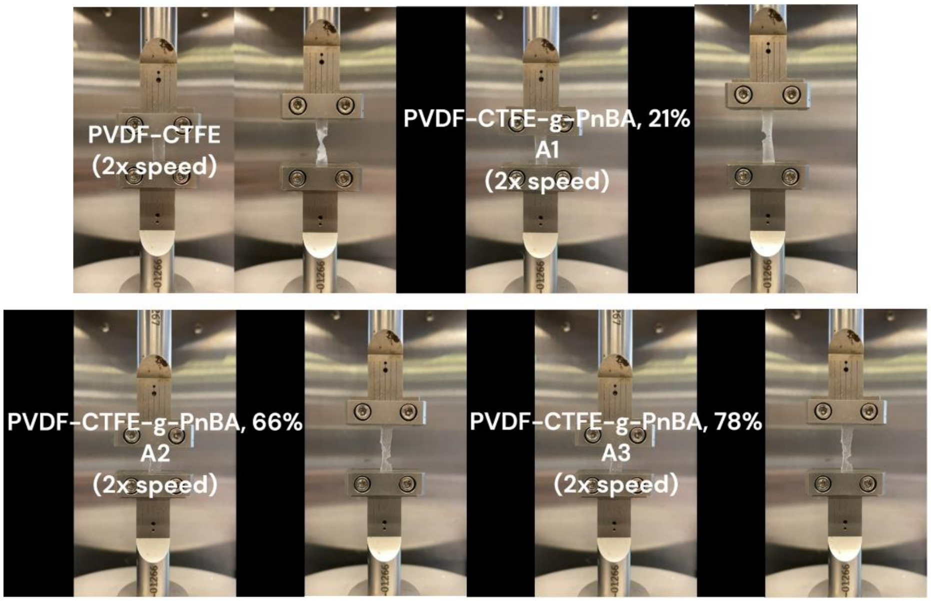

Video S1. Selected frames of the recorded tensile tests of PVDF-CTFE and PVDF-CTFE-g-PnBA (samples A1, A2, A3).

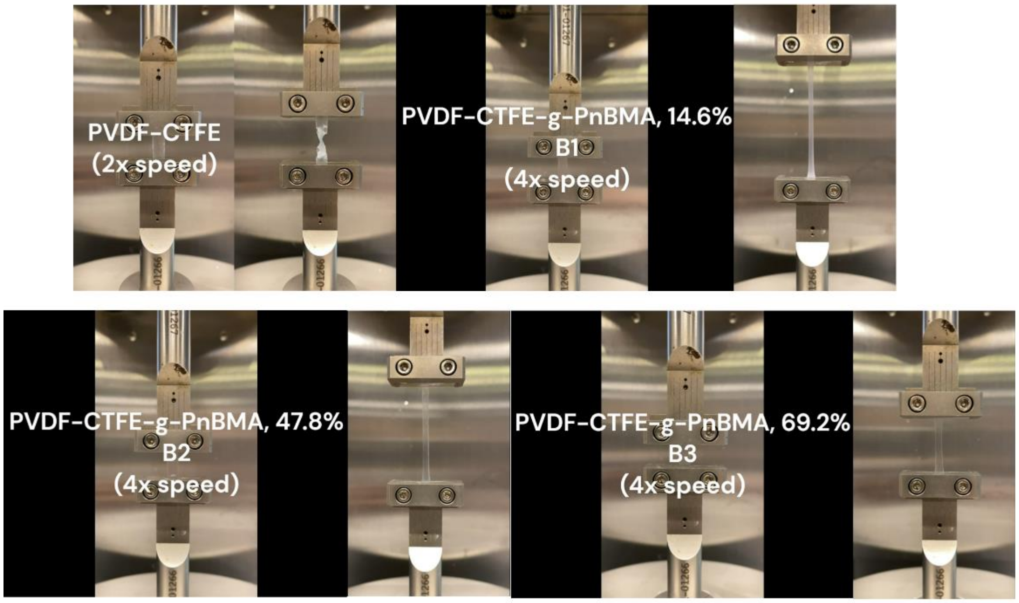

Video S2. Selected frames of the recorded tensile tests of PVDF-CTFE and PVDF-CTFE-g-PnBMA (samples B1, B2, B3).

References.

(1) Kumar, A.; Brown, S. C.; Donlan, M. E.; Meier, B. U.; Jeffs, P. W. Optimization of Two-Dimensional NMR by Matched Accumulation. *J. Magn. Reson. 1969* **1991**, *95* (1), 1–9. https://doi.org/10.1016/0022-2364(91)90320-S.

(2) Twum, E. B.; Gao, C.; Li, X.; McCord, E. F.; Fox, P. A.; Lyons, D. F.; Rinaldi, P. L. Characterization of the Chain-Ends and Branching Structures in Polyvinylidene Fluoride with Multidimensional NMR. *Macromolecules* **2012**, *45* (13), 5501–5512. https://doi.org/10.1021/ma300835s.

(3) Gong, H.; Zhang, X.; Zhang, Y.; Zheng, A.; Tan, S.; Zhang, Z. Chemical Composition Characterization of Poly(Vinylidene Fluoride-Chlorotrifluoroethylene)-Based Copolymers with F–H Decoupled 1 H NMR. *RSC Adv.* **2016**, *6* (79), 75880–75889. https://doi.org/10.1039/C6RA11757K.

(4) Daems, N.; Milis, S.; Verbeke, R.; Szymczyk, A.; P. Pescarmona, P.; J. Vankelecom, I. F. High-Performance Membranes with Full pH-Stability. *RSC Adv.* **2018**, *8* (16), 8813–8827. https://doi.org/10.1039/C7RA13663C.

(5) Bai, H.; Wang, X.; Zhou, Y.; Zhang, L. Preparation and Characterization of Poly(Vinylidene Fluoride) Composite Membranes Blended with Nano-Crystalline Cellulose. *Prog. Nat. Sci. Mater. Int.* **2012**, *22* (3), 250–257. https://doi.org/10.1016/j.pnsc.2012.04.011.

(6) Nallasamy, P.; Mohan, S. Vibrational Spectroscopic Characterization of Form II Poly(Vinylidene Fluoride). *IJPAP Vol4311 Novemb. 2005* **2005**.

(7) Chamakh, M. M.; Mrlík, M.; Leadenham, S.; Bažant, P.; Osička, J.; AlMaadeed, M. A. A.; Erturk, A.; Kuřitka, I. Vibration Sensing Systems Based on Poly(Vinylidene Fluoride) and Microwave-Assisted Synthesized ZnO Star-Like Particles with Controllable Structural and Physical Properties. *Nanomaterials* **2020**, *10* (12), 2345. https://doi.org/10.3390/nano10122345.

(8) Kawasaki, A.; Furukawa, J.; Tsuruta, T.; Wasai, G.; Makimoto, T. Infrared Spectra of Poly(Butyl Acrylates). *Makromol. Chem.* **1961**, *49* (1), 76–111. https://doi.org/10.1002/macp.1961.020490108.

(9) Pekel, N.; Güven, O. Fourier Transform Infrared–Photoacoustic Spectroscopy of Poly (N-Butyl Methacrylate) Adsorbed from Solution on Alumina. *J. Appl. Polym. Sci.* **1998**, *69* (8), 1669–1674. https://doi.org/10.1002/(SICI)1097-4628(19980822)69:8<1669::AID-APP20>3.0.CO;2-O.
